# Knowledge, Use, and Barriers to Electrical Stimulation in Upper Limb Stroke Therapy Among German Therapists: A Cross-Sectional Survey

**DOI:** 10.1155/np/4697720

**Published:** 2025-09-24

**Authors:** Sarah Tenberg, Lutz Vogt, Steffen Müller, Daniel Niederer

**Affiliations:** ^1^Department of Computer Science/Therapeutic Sciences, Trier University of Applied Sciences, Trier, Germany; ^2^Department of Sports Medicine and Exercise Physiology, Goethe-University Frankfurt, Frankfurt am Main, Germany; ^3^Department of Sports Medicine and Exercise Physiology, Institute of Occupational, Social and Environmental Medicine, Goethe University Frankfurt, Frankfurt am Main, Germany

**Keywords:** electrical stimulation, Germany, occupational therapists, physical therapists, stroke rehabilitation, upper limb, Usage

## Abstract

**Background and Purpose:** Functional electrical stimulation (FES) is an effective therapeutic method for improving upper limb motor function after stroke, yet its usage among occupational and physical therapists in Germany remains uncertain. The aim of the study is to investigate the knowledge of, frequency of use, and barriers to electrical stimulation use in stroke rehabilitation.

**Methods:** An online survey was conducted among German occupational and physical therapists working with stroke patients. Data were analyzed for frequency distributions, and associations between electrical stimulation usage and individual/organizational factors were assessed using Chi-Square or Fisher's exact tests.

**Results:** A total of *n* = 111 participants completed the survey (57 occupational and 54 physical therapists). Almost half (45%) reported regular electrical stimulation use, with 57% wanting to increase it. Use was higher among therapists with additional training (85% vs. 44%, *p*=0.041), belief in electrical stimulation effectiveness during acute (87% vs. 59%, *p*=0.041) and early subacute stages (81% vs. 47%, *p*=0.027), sufficient time (78% vs. 60%, *p* < 0.001), and device access (80% vs. 44%, *p*=0.006). Therapists with over 10 years of experience used electrical stimulation less frequently (*p* < 0.001).

**Conclusion:** Although electrical stimulation shows promise in rehabilitation, further research is needed to assess the resources—such as time, equipment, and therapist training—required for its effective integration.

## 1. Introduction

Electrical stimulation combined with task-specific training is likely one of the most effective therapies in individuals with arm paresis after stroke [1]. Among the different electrical stimulation therapies, functional electrical stimulation (FES) is effective to improve upper extremity activity [2] and seems to be the most effective to improve motor function [3].

When voluntary movement is minimal in the first months after stroke, most treatment guidelines specifically recommend FES, inter alia, in combination with motor training [4–7]. The German guideline provides an open recommendation (recommendation grade 0—“may be considered”) for the use of neuromuscular electrical stimulation of the shoulder muscles to prevent subluxation. It also suggests using it for wrist and finger extensors to promote selective movement and arm function. Additionally, FES is recommended to induce grasp and release, as well as for shoulder and elbow movements [4].

In contrast to the current recommendations, an investigation showed that 65% of physical therapists in Canada reported that they “never” or “rarely” used FES for improving arm function in individuals with stroke [8]. As barriers, a lack of access to resources, such as time, equipment, and training was named. In addition, the evidence supporting the use of FES in individuals with stroke was not known by more than 40% [8]. In Australia, only 52% of physical and occupational therapists use FES to improve upper limb activities in individuals with stroke [9]. The frequency is higher in therapists who have received electrical stimulation training, 62.5% of whom use FES in their practice.

Implementing research into clinical practice is important, but translating evidence into clinical practice takes a long time [10]. With regard to the use of technology in stroke rehabilitation, lack of knowledge and training and access to equipment were identified as key factors in the lack of implementation [11]. Since electrical stimulation should be applied by occupational and physical therapists working in stroke rehabilitation and its use in Germany is unknown, there is a research gap compared to Canada and Australia. Therefore, the aim of this study was to investigate the current status of the use of electrical stimulation for individuals with stroke and arm paresis among occupational and physical therapists, with a special focus on Germany. In addition, to investigate potential facilitators (e.g., knowledge, additional training courses) and barriers such as access to equipment and sufficient time.

## 2. Methods

### 2.1. Study Design and Ethics

The study employed a two-phase cross-sectional design. During the initial phase, an online survey was developed, validated, and translated into German. In the second phase, the survey was distributed to and filled in by occupational and physical therapists across Germany. The study was approved by the local Ethics Committee of the University of Applied Sciences Trier (No. 12-2023) and conducted in compliance with the Declaration of Helsinki. All participants provided online informed consent before survey enrollment. The reporting of the study followed the CHERRIES and CROSS reporting guidelines [12, 13].

### 2.2. Survey Development and Validation

An initial version of the survey was developed by two physiotherapists and the first author (Sarah Tenberg) based on a comprehensive literature review and practical experience. This preliminary version was then refined in a focus group (Sarah Tenberg, Lutz Vogt, Steffen Müller, Daniel Niederer). The English version for validation can be found in Supporting Information Figure S1.

The content of the survey was expert-validated by three international experts in the field of electrical stimulation in stroke rehabilitation from Canada, Switzerland, and Australia. All three experts are physical therapists with over 15 years of practical experience in stroke rehabilitation, and they also possess scientific expertise in this field. The experts rated each question of the survey on a 3-point Likert scale as irrelevant (−1), neutral (0), relevant (1). The sum scores from all three experts were used to decide whether questions should be eliminated, retained, or adjusted. Questions with a negative rating were deleted, while questions with a positive rating were retained. Questions with a neutral score were discussed in the focus group with all authors. Additionally, experts were given the opportunity to add a comment to each question, which were also reviewed in the focus group, leading to appropriate adjustments in the survey.

The revised survey was then translated into German and pilot-tested by three German physical therapists who were not involved in the development of the questionnaire. Their feedback was incorporated into the final version of the survey (Supporting Information Figure S2). Questions not relevant to this study are highlighted in red.

The final content of the survey included five categories: (1) sociodemographics, (2) work environment, (3) training and belief about the effectiveness of electrical stimulation for individuals with stroke and arm paresis, (4) use of electrical stimulation in the therapy practice of individuals with stroke and associated arm paresis (main part of the survey), and (5) potential facilitators and barriers for the use of electrical stimulation.

The primary outcome was the frequency of use of electrical stimulation for stroke rehabilitation of arm paresis. This was measured using a 5-point Likert scale with the responses: (“How often have you used electrical stimulation in your therapeutic work with individuals with stroke and arm paresis in the last two years?”) “never,” “rarely (once a month),” “sometimes (2–4 times a month),” “often (2–4 times a week),” and “always (daily).” To assess the perceived effectiveness, a 4-point Likert scale with response options: (“In my opinion, electrical stimulation is …”) “very effective,” “rather effective,” “rather ineffective,” and “very ineffective” was used. Certainty regarding the setting of electrical stimulation devices was rated on a 5-point Likert scale using the responses (“I feel…”) “very uncertain,” “somewhat uncertain,” “neutral,” “somewhat certain,” and “very certain.” Other questions included closed-ended questions with predefined options, and for some questions, it was possible to provide an additional answer.

### 2.3. Participants and Recruitment

Inclusion criteria for the survey were the following: (1) at least 18 years of age, (2) occupational or physical therapist working in Germany, (3) at least 1% of the treated patients are individuals with stroke.

Therapists were recruited in two stages [14]. Initially, 16 key professional associations and organizations in Germany, representing the fields of electrical stimulation, neurology, physiotherapy, and occupational therapy, were contacted and asked to distribute the survey to their members. After 3 weeks, two rehabilitation facilities, two hospitals, and two occupational therapy and physiotherapy practices were randomly selected in each of the 16 federal states and asked to send the survey to their employees. Two reminders were sent for each contacted organization or institution, after 1 week and after 10 days [15].

The survey was administered using the SoSciSurvey (version 3.5.01) online platform. Data collection adhered to European data protection laws. No IP addresses or personal data that could identify an individual (name, email, phone number, or address) were recorded. The collected data will not be shared with third parties, and only the authors have access to the data.

### 2.4. Data Processing and Statistical Analysis

The survey responses were exported from SoSiSurvey to Microsoft Excel for Microsoft 365 (Version 2406, Microsoft Corporation, Redmond, Washington). Data were screened for incomplete and duplicate responses. Questionnaires containing solely demographic information or duplicates were excluded. Questionnaires were processed for analysis if they contained information for verification of the research question (e.g., information on the use of electrical stimulation in the therapy practice of individuals with stroke and arm paresis).

First, response metrics were calculated [12]. Demographic information and information about the work environment were presented descriptively. Further data were analyzed in compliance with the underlying assumptions for parametric and nonparametric tests.

For an early versus late responder analysis, the first 10 responses were compared to the last 10 responses using a Chi-Square contingency table and Fisher's exact test.

The descriptive analysis primarily involved the absolute and relative distribution of the use of electrical stimulation, including frequency, body region, and type of application. Second, confidence in the setting, such as electrode placement, use of preset protocols, and setting of custom parameters, as well as the individual setting of different stimulation parameters (intensity, frequency, pulse duration, and shape), and potential facilitators and barriers (desirable factors, access to equipment, and sufficient time) were analyzed. These aspects were visualized using histograms.

Inferential analysis included an exploratory examination of potential associations between the primary outcome, frequency of use, and training in electrical stimulation, belief in the effectiveness, therapists', and institutional characteristics using the Chi-Square test or Fisher's exact test depending on the cell distribution. The results were considered statistically significant with *α* = 5%. In order to reduce the number of cells and guarantee sufficient responses per cell for the quantitative analysis, the data were pooled “very effective” and “rather effective” were pooled into “belief in effectiveness.” “Rather ineffective” and “very ineffective” were pooled into “no belief in effectiveness.” Data were visualized using stacked bar charts.

## 3. Results/Findings

### 3.1. Response Metrics and Study Population

Participation rate was 96% (ratio of 133 visitors who agreed to participate to 139 visitors to the first survey page). The completion rate was 60% (ratio of 80 users who finished the survey to 133 users who agreed to participate). In cases where participants dropped out during the questionnaire, this mainly occurred in the section three about electrical stimulation training and knowledge of electrical stimulation (22%). 111 questionnaires fulfilled the inclusion criteria and were integrated into the analysis. Early versus late response analysis showed no differences between response time and primary outcome, frequency of use (*p*=1).

**Figure 1:**
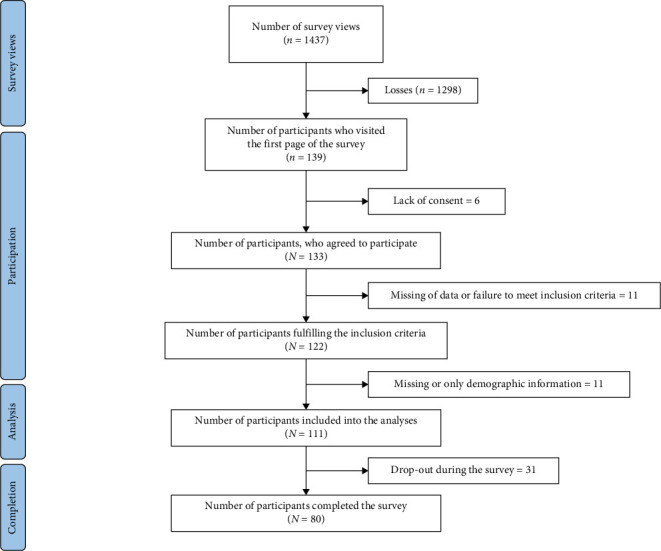
Participants flow diagram.

Participant flow can be found in Figure 1. Characteristics of the study population can be found in Table 1.

#### 3.1.1. Training and Application of Electrical Stimulation in Therapy Practice

A third (*n* = 38, 35%) of therapists reported having received electrical stimulation training during their educational program, with only one occupational therapist. Additionally, 61 therapists (58%) reported receiving additional training in electrical stimulation. The median total electrical stimulation training was 20 h (minimum: 1 and maximum: 1000).

Almost half (36, 45%) of the therapists indicated the regular use of electrical stimulation in their therapy practice (several times per month). However, 21 therapists (29%) reported never using electrical stimulation in their practice. 39 therapists (57%) would like to increase their use of electrical stimulation.

Most therapists reported using active electrical stimulation, with 47% also employing cyclic stimulation without voluntary participation. Electrical stimulation is most commonly applied to the wrist (96%).

Regarding the confidence in the setting of electrical stimulation, 45 therapists (64%) feel certain when placing electrodes, 38 (54%) when using preset stimulation protocols, and 31 (44%) when setting stimulation parameters themselves. Additionally, 51 therapists (73%) indicated individual setting of stimulation intensity for their patients, with 44 therapists (63%) setting stimulation frequency individually, 45 (64%) pulse duration, and 34 (49%) pulse shape.

The most frequently mentioned facilitator was training (in-person or online) in electrical stimulation (77%). Most therapists indicated that they have access to electrical stimulation devices (77%) and sufficient time for application (59%).

**Figure 2:**
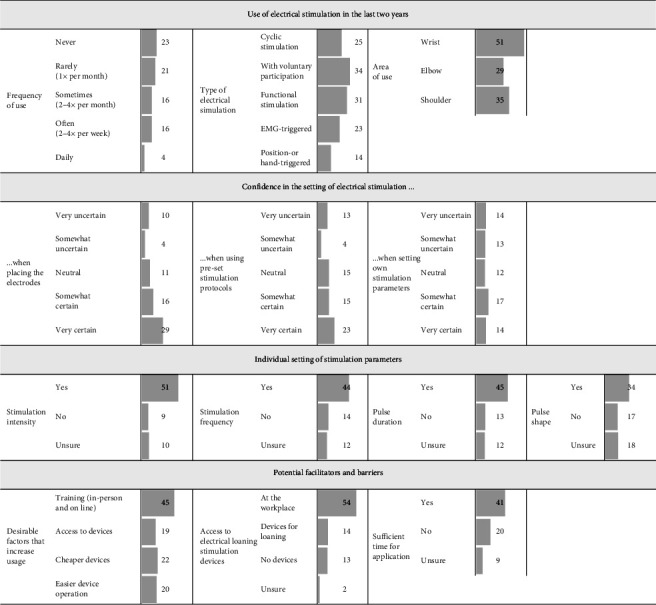
Distribution of responses regarding the use of electrical stimulation, confidence in the setting, individual settings, and potential facilitators and barriers. Numbers indicate the quantity of responses. Bars represent the relative frequencies relative to total respondents for each question. Questions regarding the types of electrical stimulation, area of use, desirable factors, and access to electrical stimulation devices allowed for multiple answers.

A detailed description with relative and absolute responses regarding the use of electrical stimulation, confidence in the settings and individual settings, as well as potential facilitators and barriers, can be found in Figure 2.

### 3.2. Explorative Analysis

**Figure 3:**
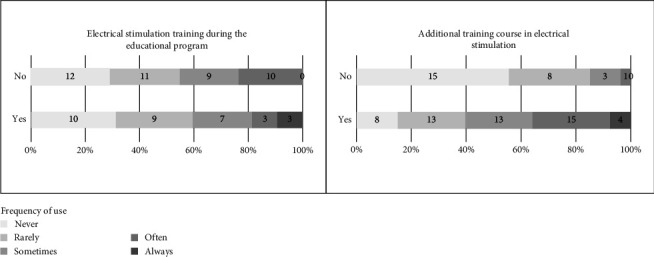
Relative and absolute respondents categorized by training received for electrical stimulation and frequency of use.

Fisher's exact test showed a significant difference between therapists who had additional training in electrical stimulation and frequency of use and those who have not (*p* < 0.001). Therapists who had received additional training reported regular use more frequently (85%) than those without such training (44%). However, no significant difference was found for training received during their educational program (*p*=0.210). The underlying descriptive results are shown in Figure 3.

**Figure 4:**
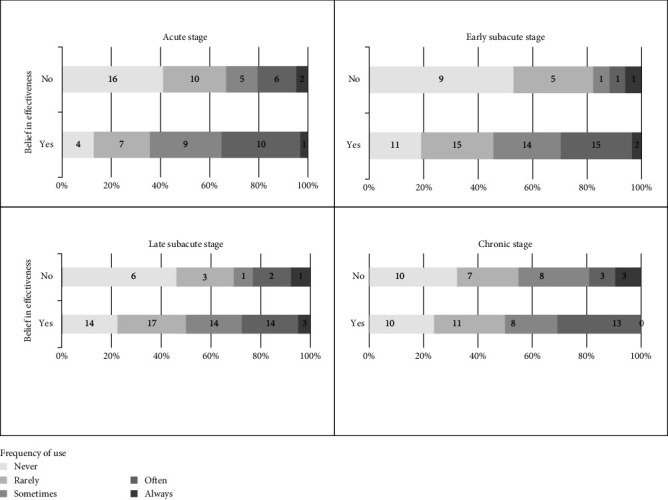
Relative and absolute respondents categorized by belief in effectiveness during different stroke stages and frequency of use of electrical stimulation.

We also found a significant difference between the belief in effectiveness during the acute stage (*p*=0.041) and the early subacute stage (*p*=0.027). Therapists who believed in the effectiveness used electrical stimulation more frequently (acute stage: 87%, subacute stage: 81%) than those who did not believe in the effectiveness (acute stage: 59%, subacute stage: 47%). No significant difference was found for the belief in effectiveness during the late subacute stage (*p*=0.397), and the chronic stage (*p*=0.069). Descriptive results can be found in Figure 4.

**Figure 5:**
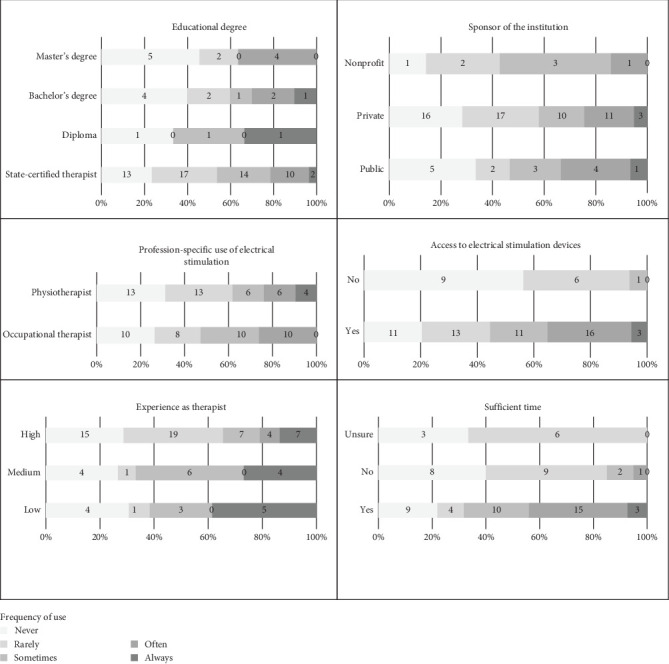
Relative and absolute respondents categorized by different therapists' and institutional characteristics, as well as frequency of use of electrical stimulation.

Regarding therapist characteristics, we found a significant difference with the frequency of use for therapists' overall experience (*p*=0.041) with a lower regular use for highly experienced therapists (>10 years), but no significant difference for educational degree (*p*=0.190) and profession (*p*=0.129). Concerning institutional characteristics, we found significant associations with the frequency of use for the indication of sufficient time (*p* < 0.001) and access to electrical stimulation devices (*p*=0.006). Therapists who reported having sufficient time used electrical stimulation more regularly (78%) than those who did not report having sufficient time (60%). Similarly, therapists with access to devices reported higher regular use (80%) than those without access (44%). No significant difference was found with regard to the type of institutional sponsor (*p*=0.802). Descriptive results are shown in Figure 5.

## 4. Discussion

The main finding of the study is that almost half of therapists regularly use electrical stimulation in their treatment of individuals with stroke and arm paresis. This frequency increases to 60% among therapists who have received additional training in electrical stimulation. Additionally, the regular use of electrical stimulation is more common among therapists who believe in the effectiveness of electrical stimulation during the acute and early subacute stroke stages, and who have sufficient time and access to devices. Experienced therapists use electrical stimulation less regularly than less experienced ones.

### 4.1. Use of Electrical Stimulation in Upper Limb Stroke Rehabilitation

In two comparable studies, 51% and 52% of participants reported using FES for upper limb training in the past 2 years, which partly aligns with our findings (regular use: 45%; use in the last 2 years: 71%) [8, 9]. Specifically, 67% of Victorian occupational therapists and 23% of physiotherapists utilized FES within this timeframe [9]. In contrast, we did not observe a difference in electrical stimulation usage frequencies between occupational and physical therapists.

It is important to note that electrical stimulation training is not included in the entry-to-practice curriculum for occupational therapists in Germany, so that additional training, the experience of colleagues, and expectations from superiors might play an important role. A key distinction between the studies is the assessment of electrical stimulation usage in the last 2 years. While the Victorian study [9] only inquired about the usage of FES over the past 2 years, the Canadian study reported the frequency of use as a percentage of therapy time [9]. In contrast, we indicated the frequency of use in times per month or week. Given that our study found a meaningful difference between regular usage (45%) and usage in the last 2 years (71%), it is likely that regular usage in Victoria is significantly lower. Additionally, the range of “rarely” in the Canadian study is quite broad, as 1% of the time equates to 4.8 min per day for a full-time position, while 20% corresponds to 96 min per day, suggesting that it is used quite regularly. As the positive effects of electrical stimulation have been proven in intervention studies lasting several weeks [2], and one-time usage is unlikely to be effective, we decided to use regular usage (multiple times per month) as the primary outcome. However, this makes comparison between the surveys challenging. An international comparison of usage would be interesting in this context.

Electrical stimulation is most frequently applied to the wrist, aligning with the observation that hand and finger motor functions are often more severely affected than the proximal upper extremity [16, 17]. Almost two-thirds of therapists use electrical stimulation with voluntary patient participation, and over half employ it during functional tasks, which aligns with evidence-based use of electrical stimulation [1, 3]. Nonetheless, almost half of the therapists still use passive stimulation, for which evidence is lacking [1]. We did not, however, inquire about the specific goals for which this method is applied.

Interestingly, therapists with over 10 years of experience use electrical stimulation less frequently than those with less experience. This may be related to factors such as age or training, with older therapists possibly having a less favorable attitude toward technology, while younger therapists may be more accustomed to it [18].

### 4.2. Barriers for Application

Implementation barriers can occur at various levels: individual (knowledge and skills), team (e.g., teamwork and roles), organizational (time and resources), and guideline (contradictory attitudes and beliefs towards stroke guidelines) levels [19].

At the individual level, therapists exhibit higher levels of uncertainty when adjusting their own stimulation parameters compared to using preset stimulation protocols. Notably, 13% of therapists do not customize stimulation intensity for their patients, and 14% are uncertain about their adjustments. Even higher uncertainty rates were observed for stimulation frequency, pulse duration, and pulse shape. Therapy barriers can include a lack of confidence and staff knowledge regarding electrical stimulation [20]. Furthermore, lack of knowledge and skill is consistently reported as one of the most common barriers [21].

A significant proportion of therapists investigated in our study do not believe in the effectiveness of electrical stimulation during different stroke stages: 56% during the acute stage, 23% during the early subacute stage, 17% during the late subacute stage, and 21% during the chronic stage. This highlights a notable knowledge gap among therapists, despite increasing evidence supporting its efficacy, particularly in the acute and subacute stages [1, 22, 23].

Remarkably, additional training in electrical stimulation has been shown to increase its use, whereas training during entry-level programs showed less impact. This underscores the importance of the type of training. Menon et al. [24] recommend an active, multicomponent education approach for better implementation strategies among physical therapists.

At the institutional level, access to devices and sufficient time emerge as main barriers, consistent with previous findings [8]. In addition, time pressure in delivering electrical stimulation may also be a primary barrier for therapists to adopt electrical stimulation [20]. Despite the majority of therapists in our study reporting sufficient time, perceived time constraints could hinder the use of electrical stimulation, especially among those who feel they have inadequate time. Moreover, most therapists' workplaces lack equipment for loaning devices to patients. Additionally, while the influence of the German Guideline for Stroke Rehabilitation in Arm Paresis has not been investigated, the open recommendation (grade 0) [4] may represent a further barrier for therapists to use electrical stimulation.

### 4.3. Future Research Implications

Our study investigated the usage of electrical stimulation at the therapist level. Future research should focus on the utilization of electrical stimulation at the institutional level, assessing the overall utilization of electrical stimulation within institutions. In addition, investigating the contextual factors that influence the implementation of electrical stimulation and explaining the variability at both the therapist and institutional levels would be valuable.

Additionally, implementation research is needed to explore various training approaches, such as e-learning courses, on-the-job training, and the development of electrical stimulation specialists. Moreover, examining the impact of guideline recommendations through cross-country comparisons could provide valuable insights into the broader adoption and effectiveness of electrical stimulation practices.

### 4.4. Limitations

We only surveyed a small percentage of therapists in Germany. The proportion of male participants in our sample is high, which may not accurately represent the demographics of occupational and physical therapists practicing stroke rehabilitation in Germany. Additionally, a significant number of respondents work in the private sector. We obtained survey responses from 14 of the 16 states in Germany, which enhances the generalizability of our findings. However, the small and possibly unrepresentative sample limits the conclusions that can be drawn from this study.

In summary, both occupational and physical therapists in Germany use electrical stimulation to treat arm paresis after stroke, though most therapists wish to increase its clinical application. In particular, enhanced training opportunities, both in-person and online, along with adequate resources—such as access to electrical stimulation devices and sufficient time for therapists—may support the broader implementation of electrical stimulation. Further research is needed to evaluate these resources and develop specific implementation strategies for the effective integration of electrical stimulation in stroke rehabilitation.

## 5. Implications for Occupational and Physiotherapy Practice

The integration of technology into therapy has enhanced treatment effectiveness but also introduced new challenges, requiring therapists to continuously expand their knowledge. Electrical stimulation devices, in particular, are complex, and many therapists feel uncertain about their use, often not utilizing them to their full potential. Our study suggests that targeted training—both on-the-job and online—could significantly increase the adoption and effective use of electrical stimulation. However, limited time and financial resources pose barriers to widespread training. A practical solution could be to designate specialists for specific technologies, such as electrical stimulation, allowing general practitioners to refer patients when necessary. Additionally, e-learning courses provide a flexible and cost-effective option for self-directed training [21].

Despite the potential benefits of FES in improving motor function and activities in individuals with stroke-related arm paresis, our findings indicate that it is often applied passively in clinical practice. A simple yet effective way to improve the use of electrical stimulation is to encourage active patient engagement during stimulation. Greater emphasis on interactive methods, such as EMG-triggered or position-triggered electrical stimulation, could further optimize patient outcomes.

## Figures and Tables

**Table 1:** Characteristics and work environment of the study population.

*N* = 111		Mean	Standard deviation
Age (years)		42	13
Experience with individuals with stroke (years)		15	10

		**Numbers (*n*)**	**Frequency (%)**

Profession	Occupational therapist	57	51%
	Physical therapist	54	49%

Gender	Male	76	68%
	Female	33	30%
	NA	2	2%

Education	State-certified therapists	76	68%
	Academic (Diplom/BA/MA)	35 (3/19/13)	32 (3/17/12)%

Experience as therapist	<5 years	16	14%
	5–10 years	21	19%
	>10 years	74	67%

Health care institution	Acute care/hospital (Inclusive rehabilitation/therapy practice)	20 (6/6)	18 (5/5)%
	Inpatient/outpatient rehabilitation	27	24%
	Therapy practice (Inclusive community care/care facility/acute/rehab)	64 (13/7/1/2)	58 (11/6/1/2)%

Funding of the health care institution	Private	81	73%
	Public	21	19%
	Nonprofit	8	7%
	NA	1	1%

Primary area of practice	Neurology	69	62%
	Orthopedics	25	23%
	Geriatrics	4	4%
	Padeatrics	7	6%
	Other/more than one area	6	5%

Age group of the patients	≥18 years	59	53%
	<18 years	11	10%
	All ages	39	35%
	NA	2	2%

Relative proportion of individuals with stroke among all patients	1%–20%	45	41%
	21%–40%	23	21%
	41%–59%	11	10%
	60%–79%	17	15%
	≥80%	15	14%

Represented states out of a possible 16 (missing ones)		14 (Bremen, Hamburg)	88%

## Data Availability

The data that support the findings of this study are available from the corresponding author upon reasonable request.

## Supporting Information

Supporting Information Figure S1: Validation version of the survey. Figure S2: Validated German version of the survey.
