# Supplementary material for: Knowledge, Use, and Barriers to Electrical Stimulation in Upper Limb Stroke Therapy Among German Therapists: A Cross-Sectional Survey
Source: Neural Plast. 2025 Sep 24;2025:4697720. doi: 10.1155/np/4697720 (PMC12488299; doi:10.1155/np/4697720)

**Supplemental Materials**

Knowledge, use, and barriers to electrical stimulation in upper limb stroke therapy among German therapists: a cross-sectional survey

Tenberg et al. 2025

Figure S1: Validation version of the survey


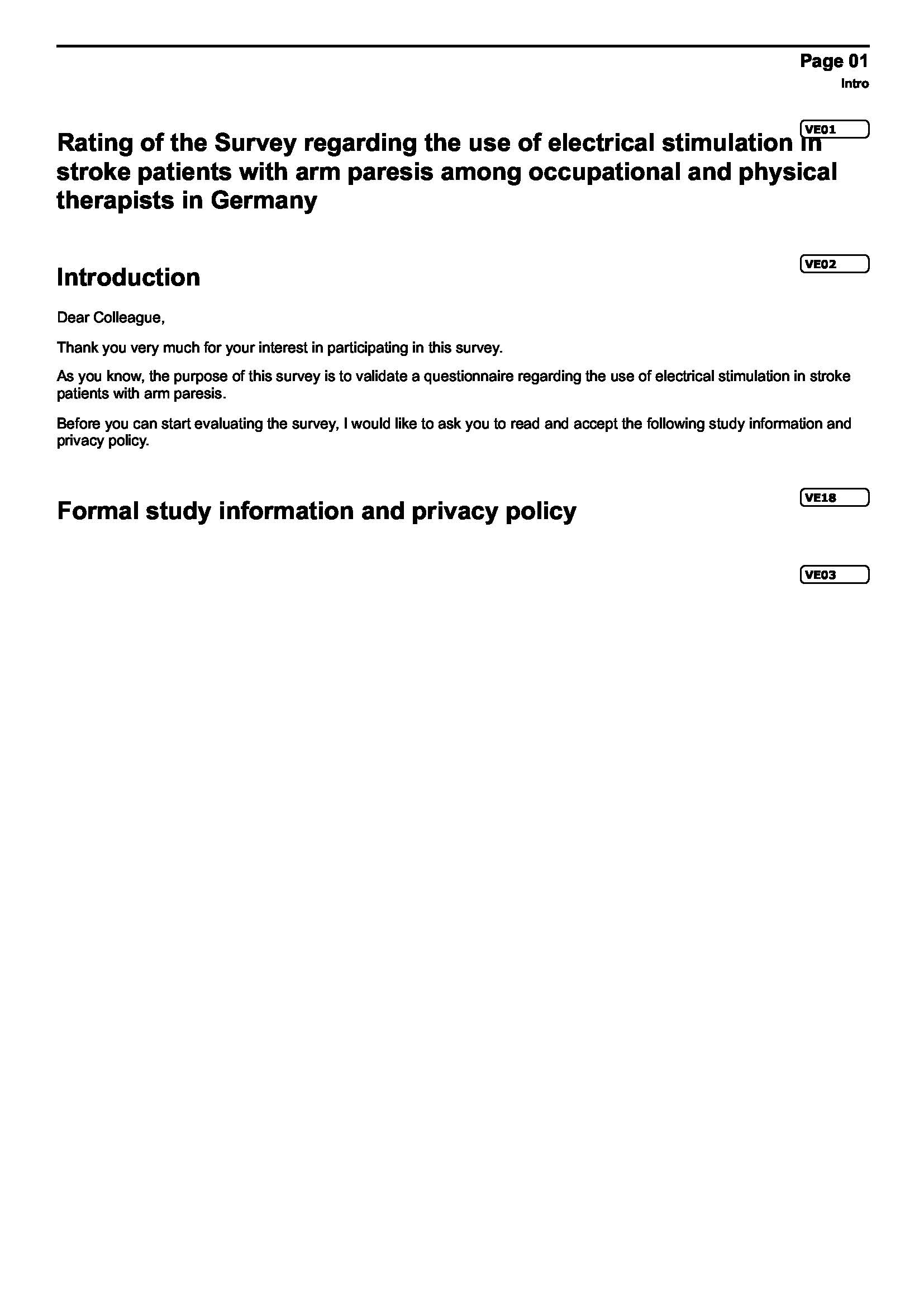


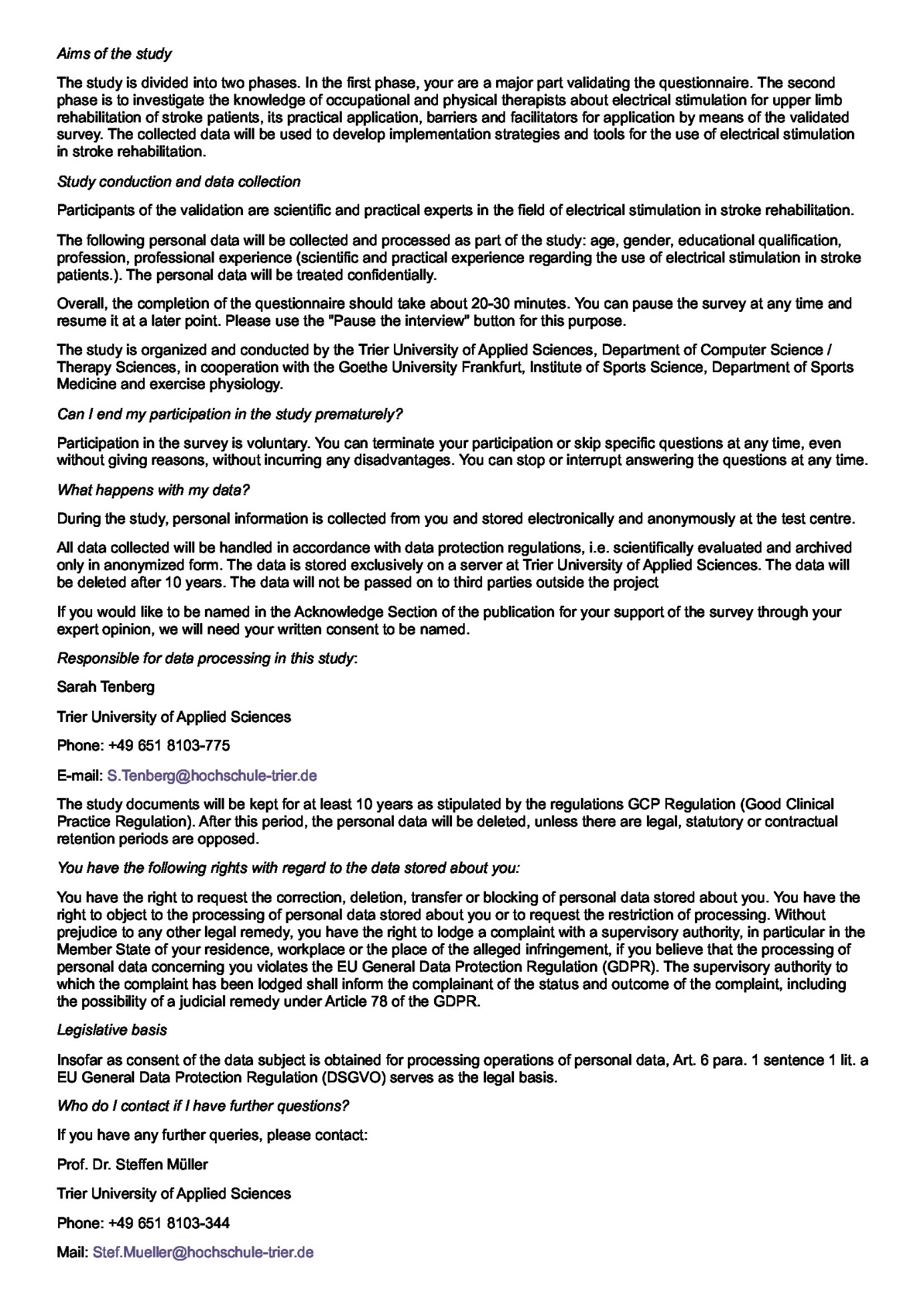


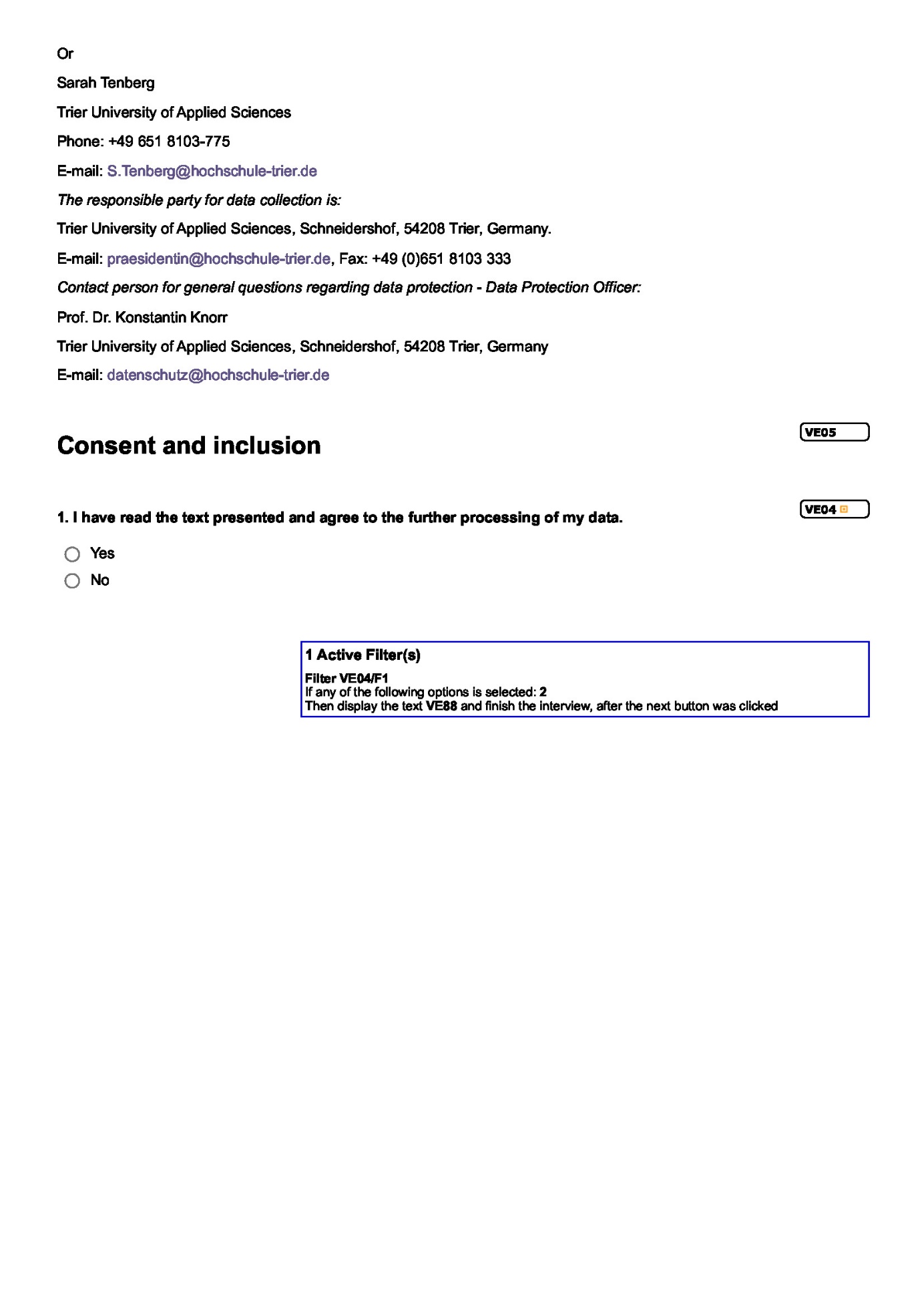


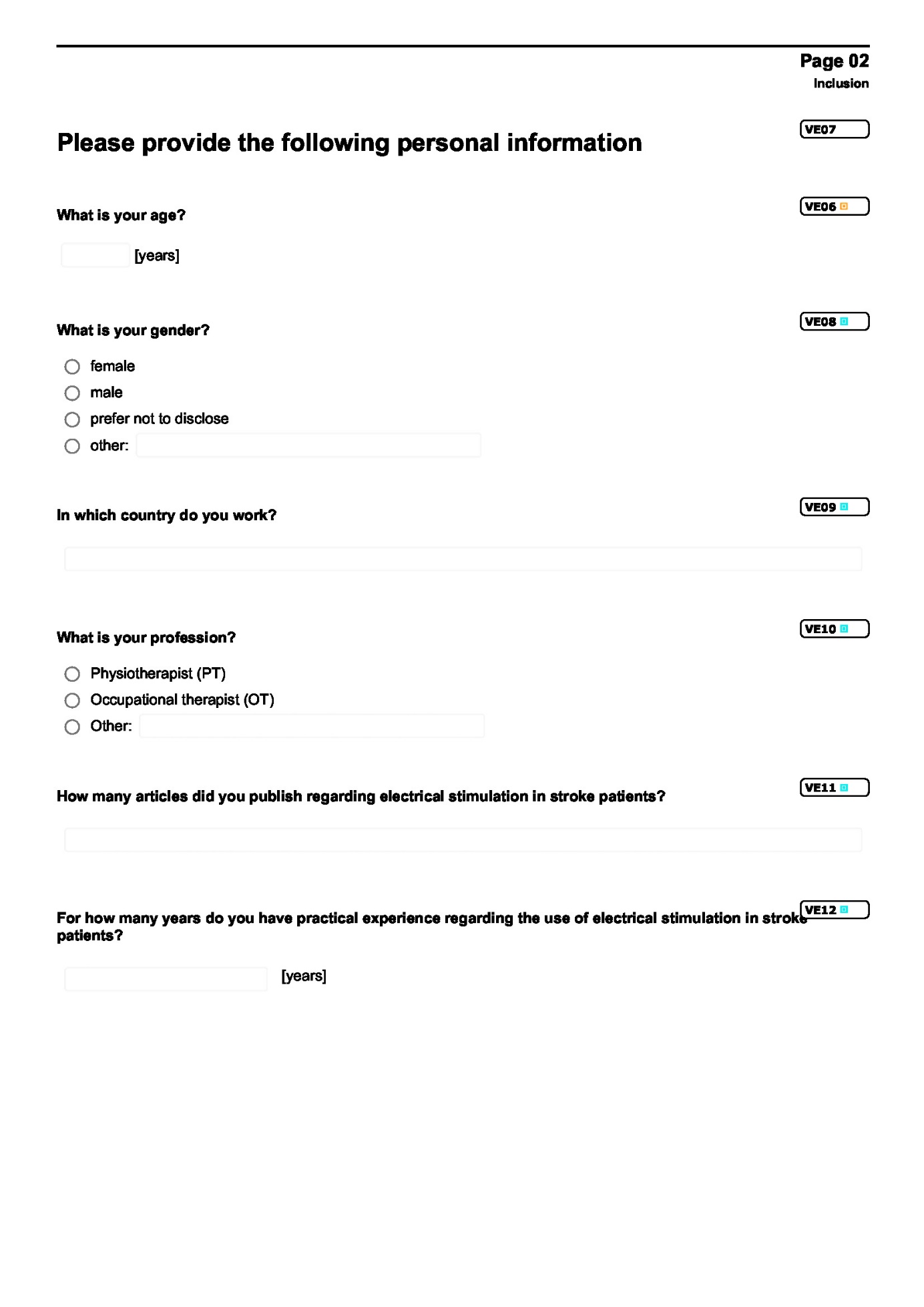


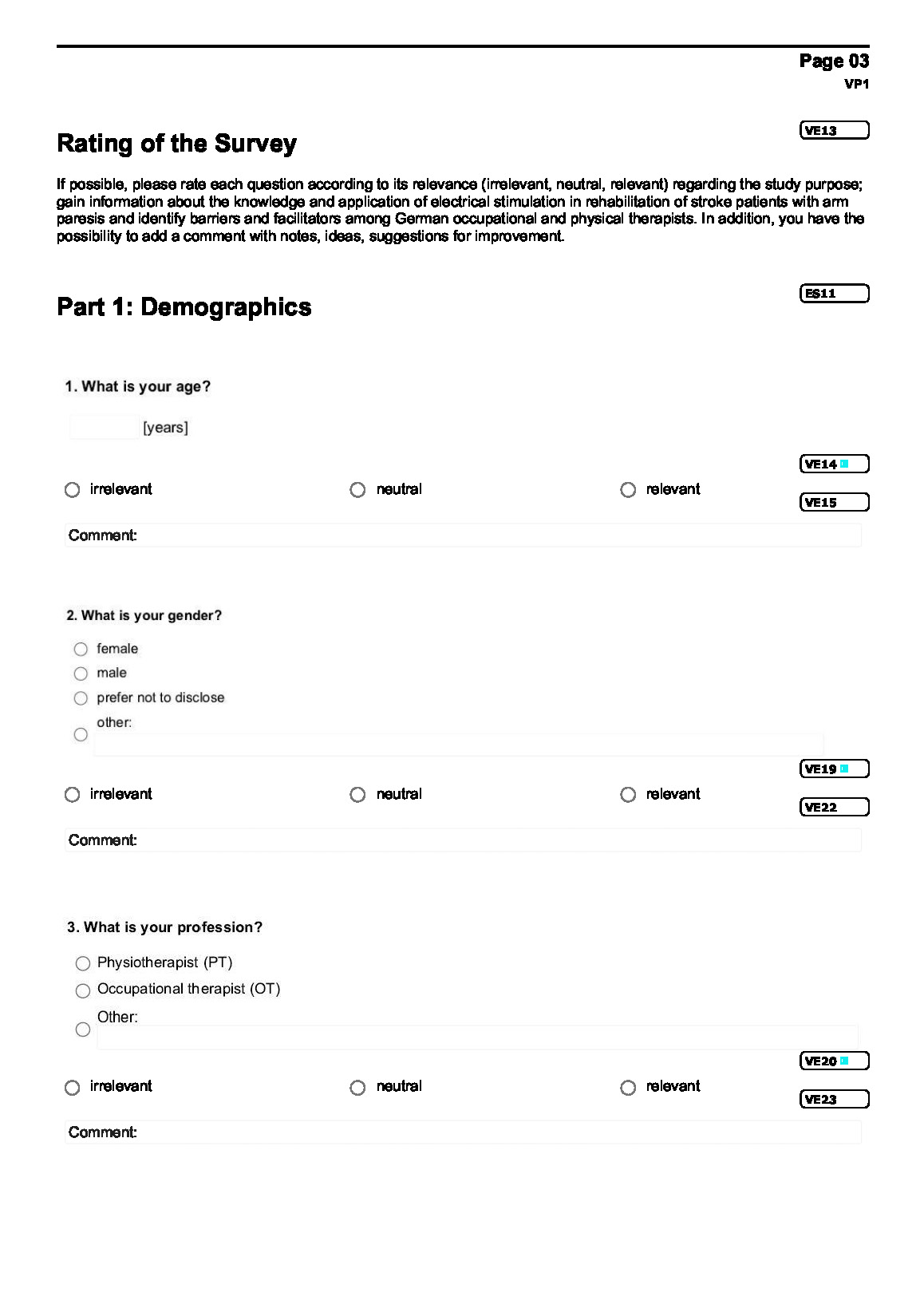


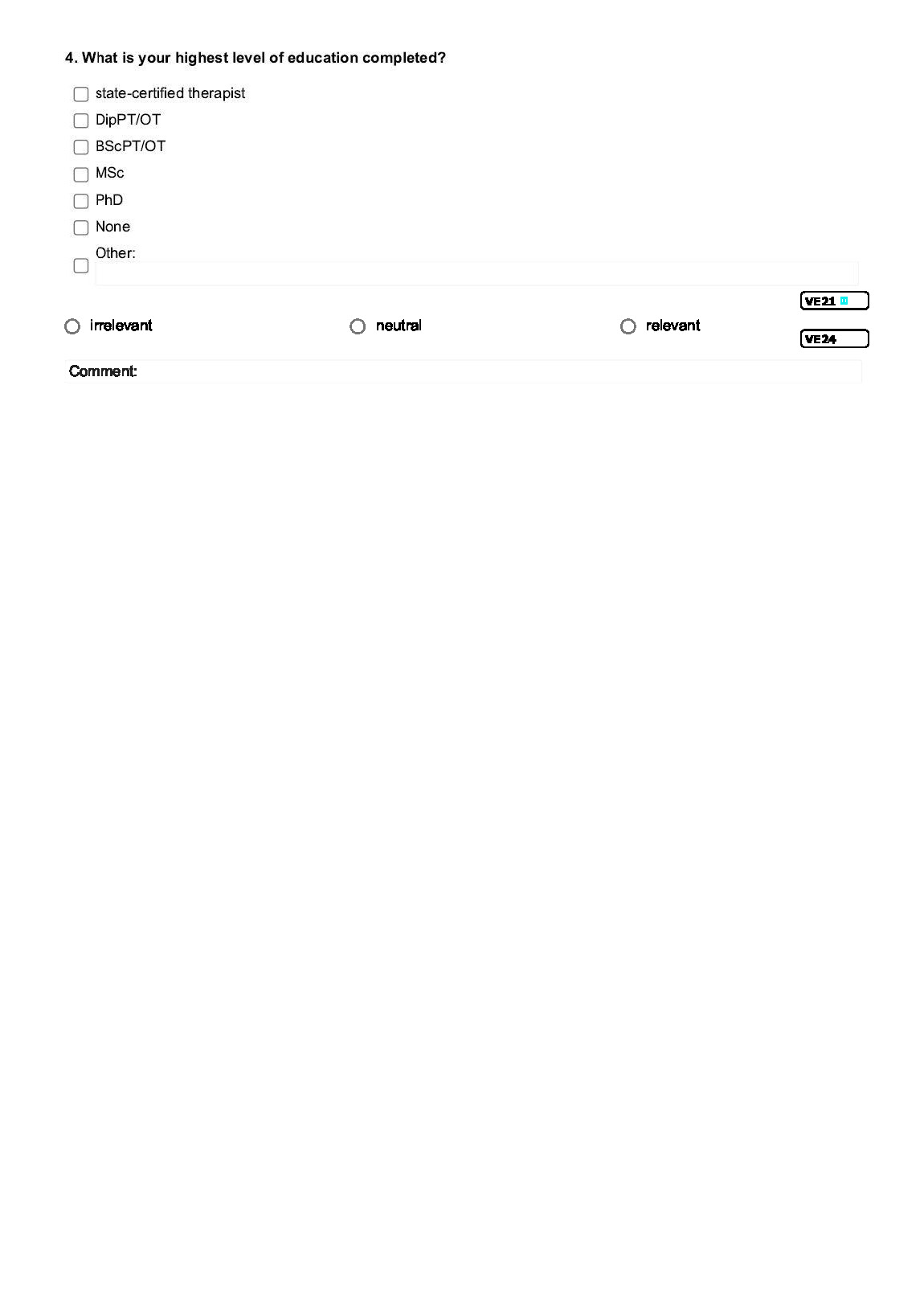


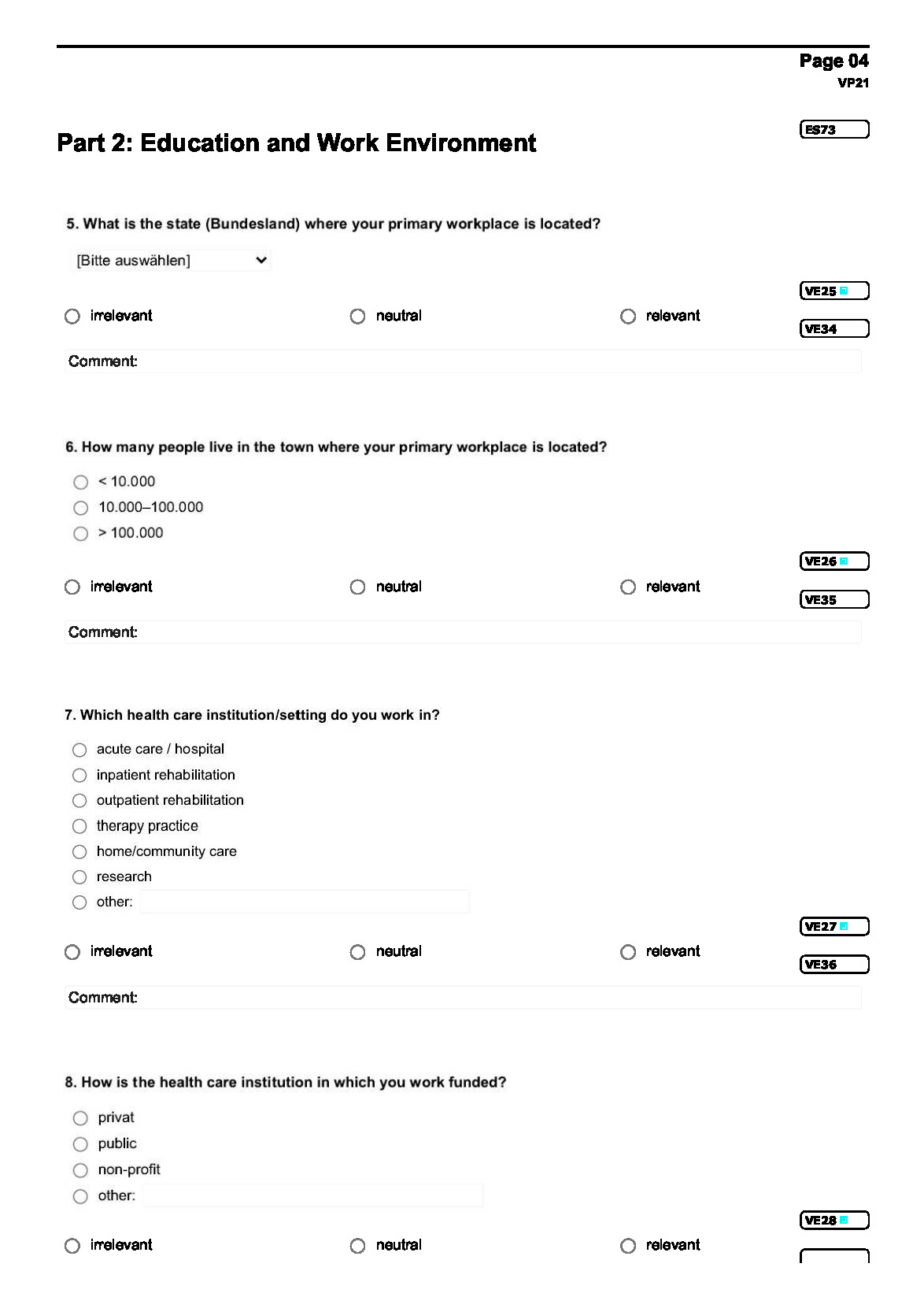


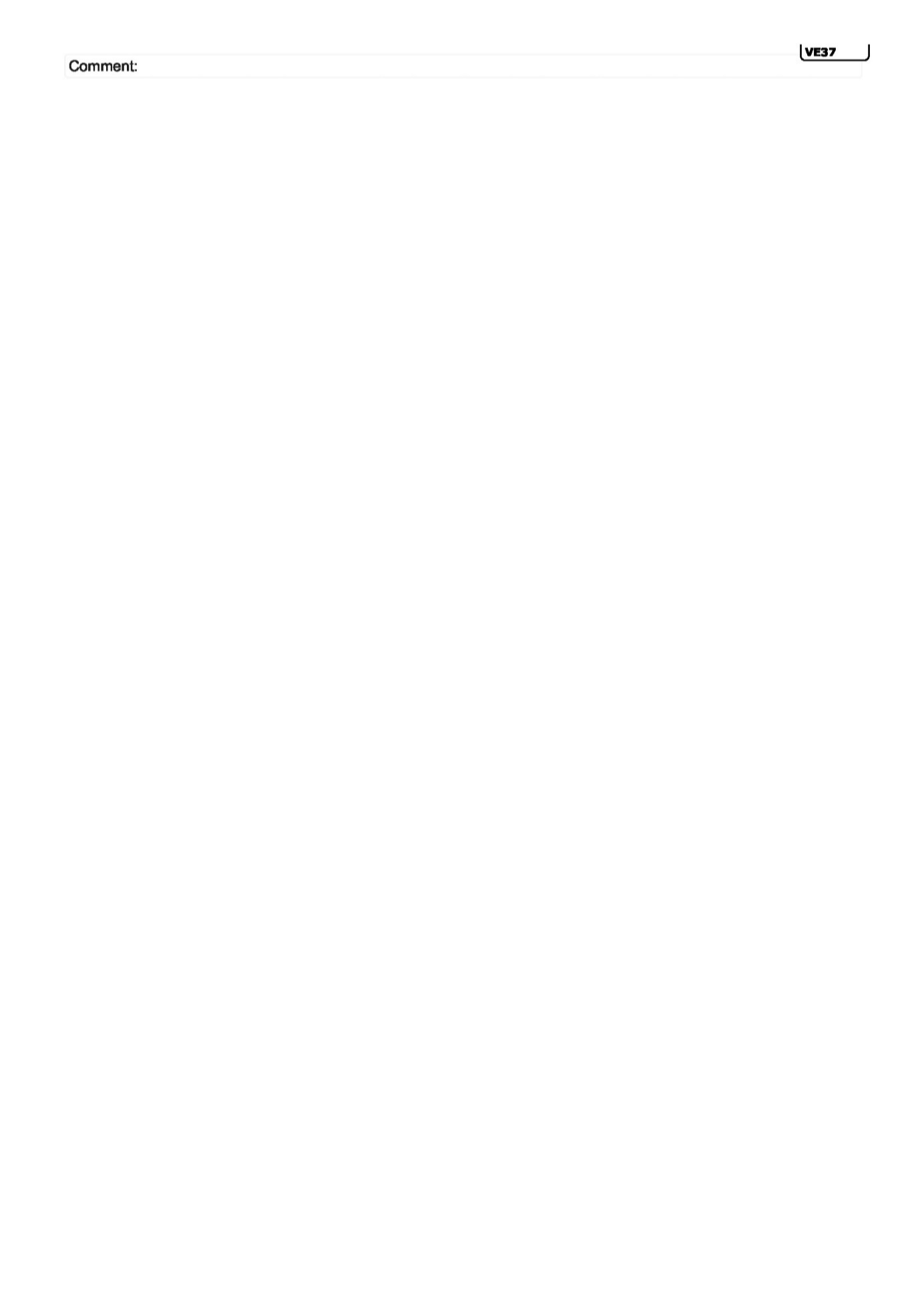


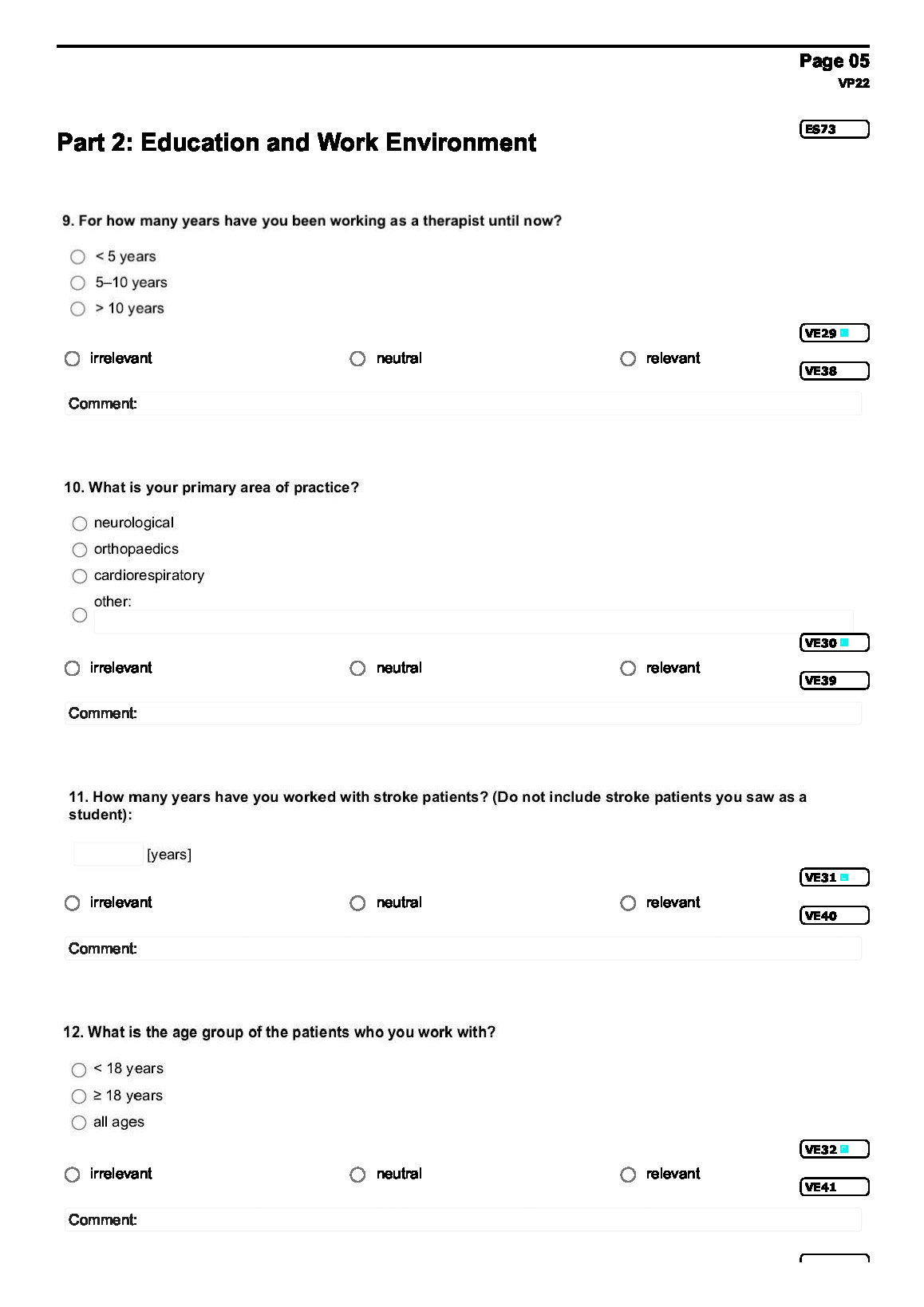


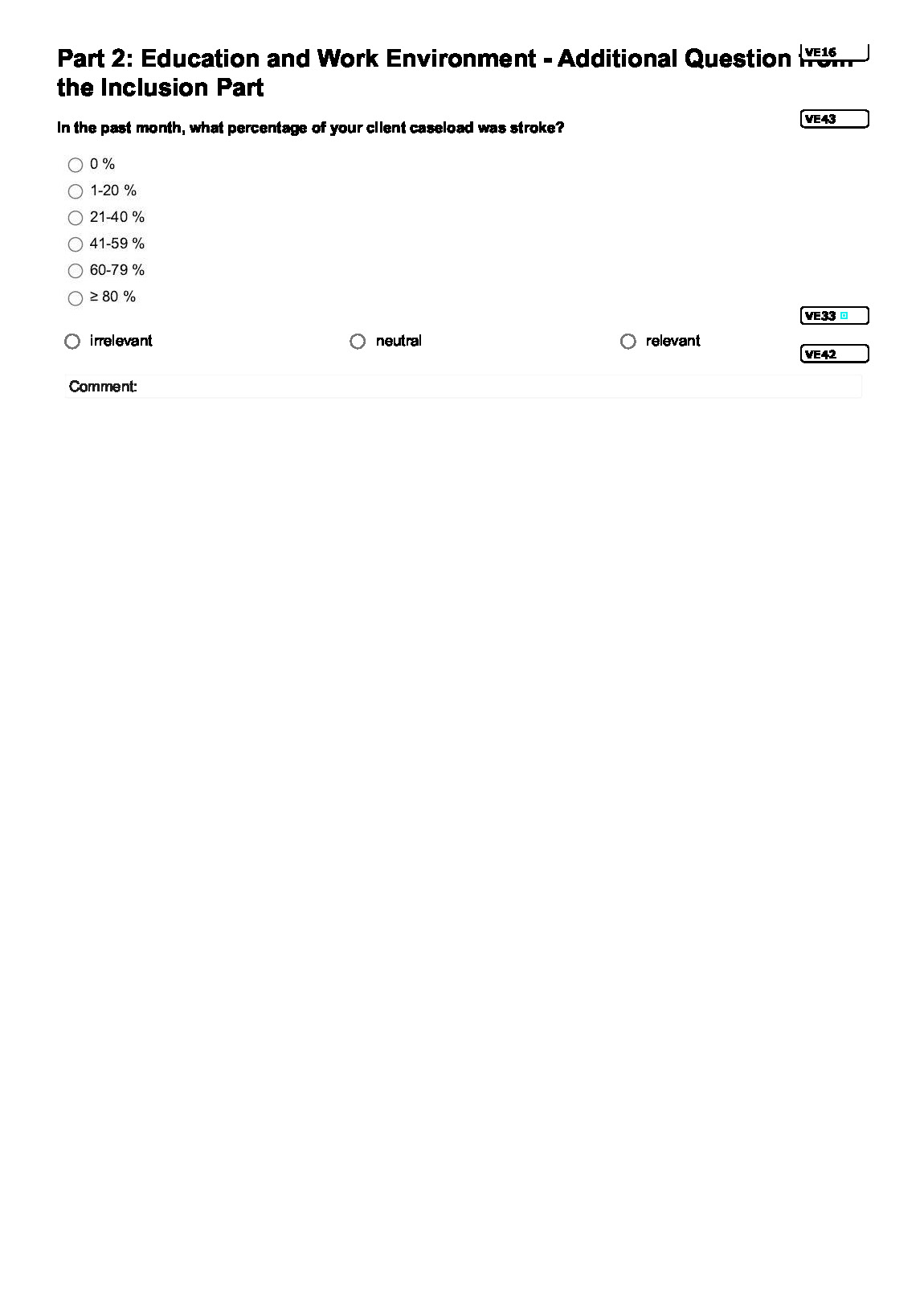


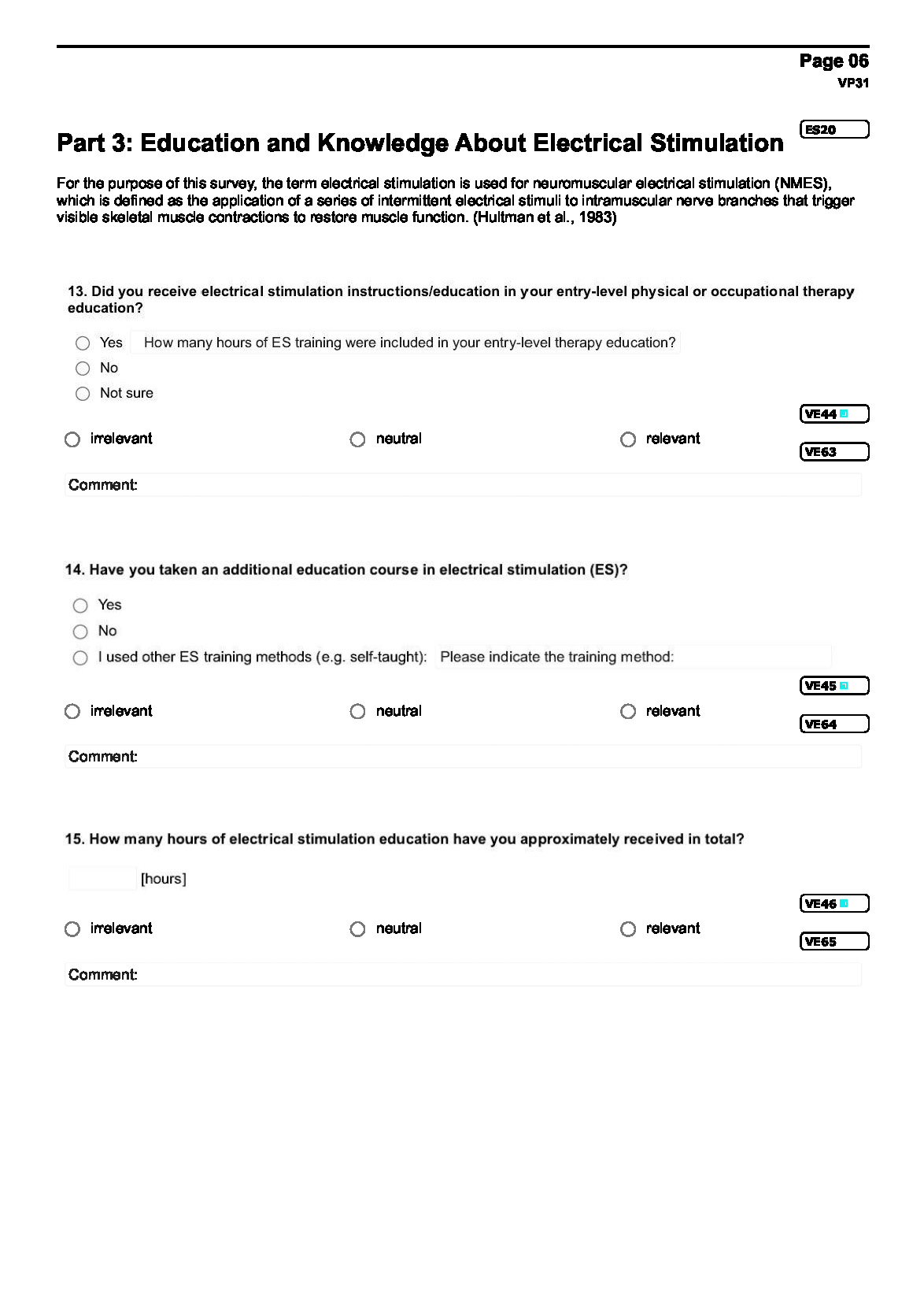


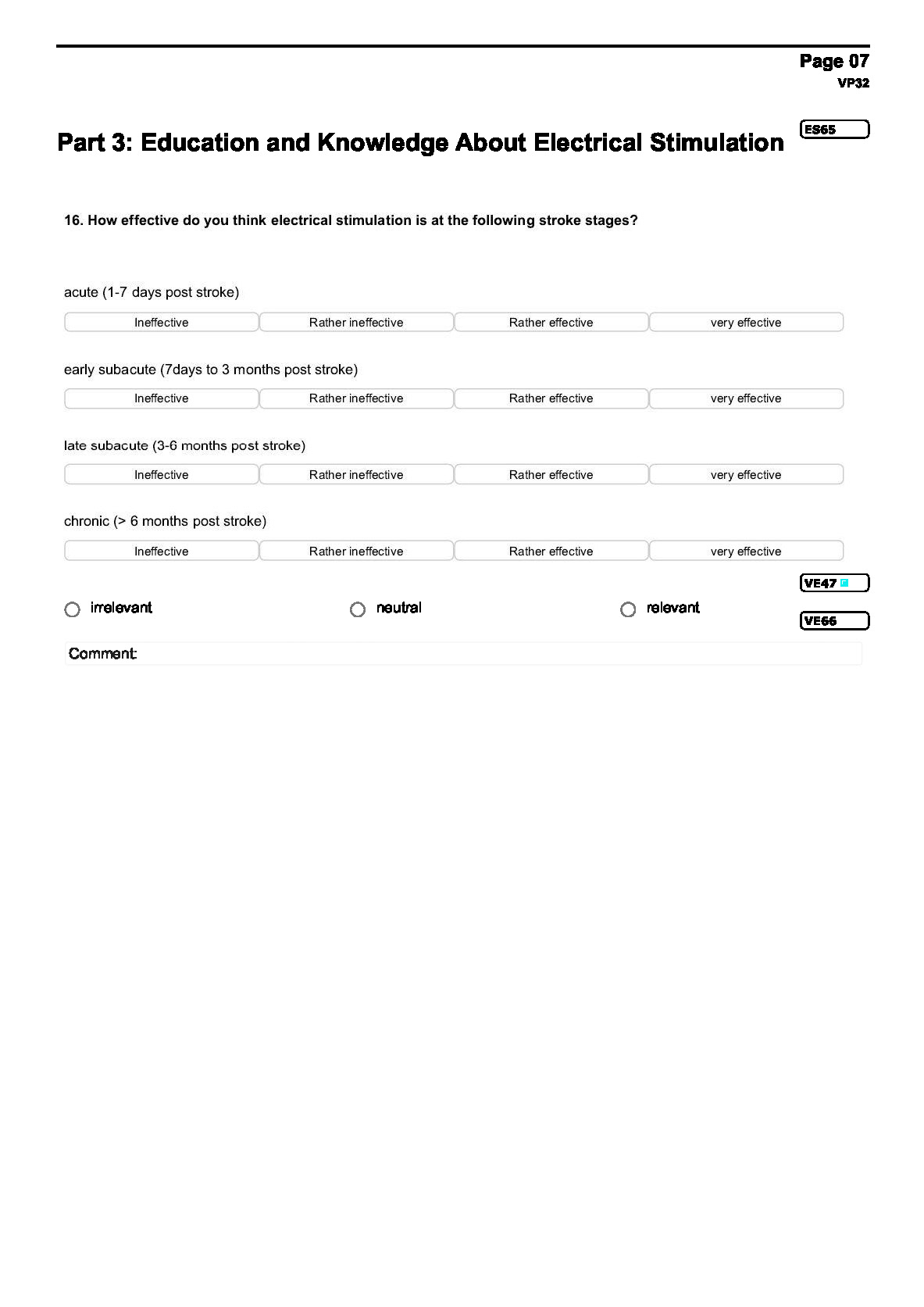

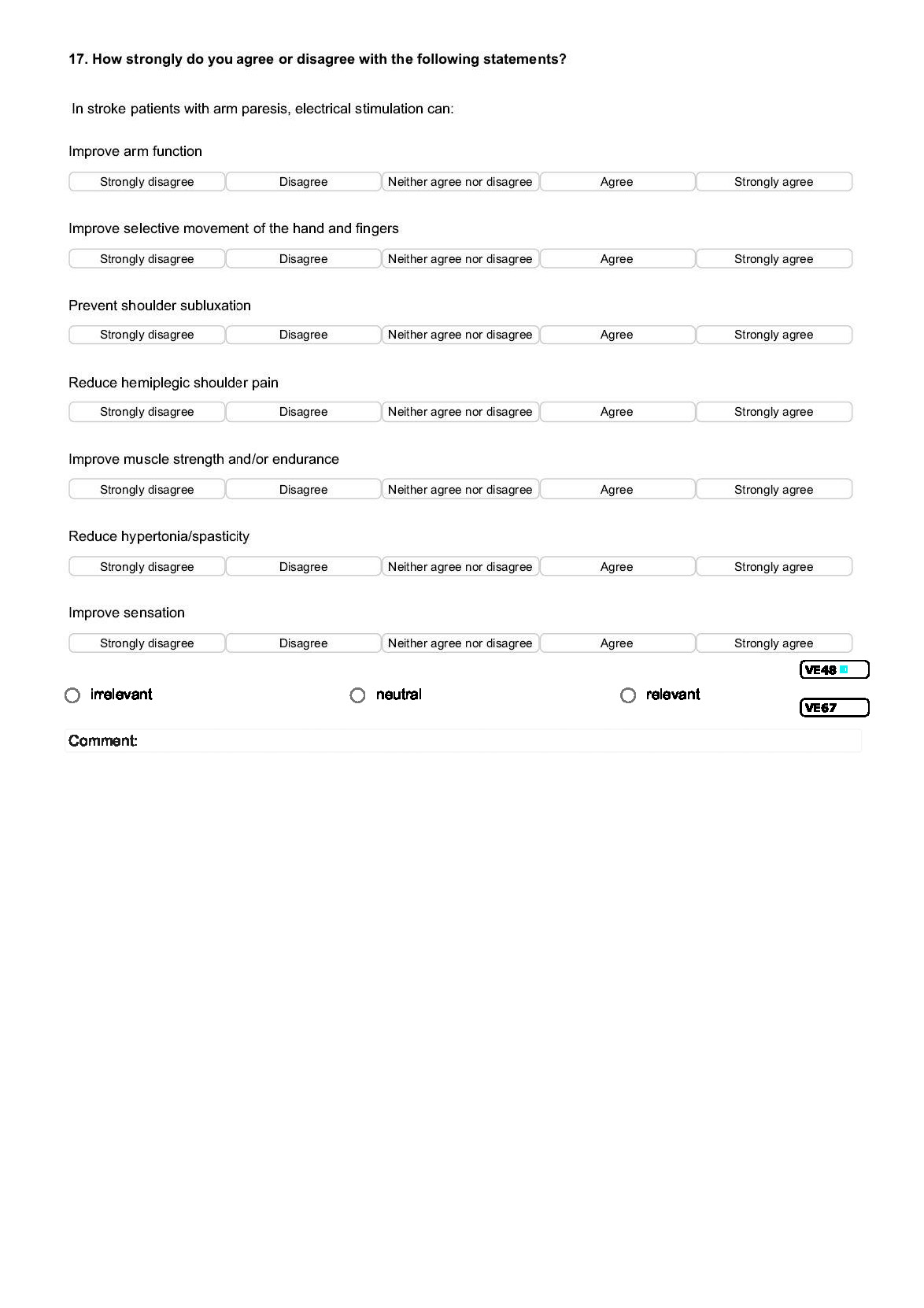

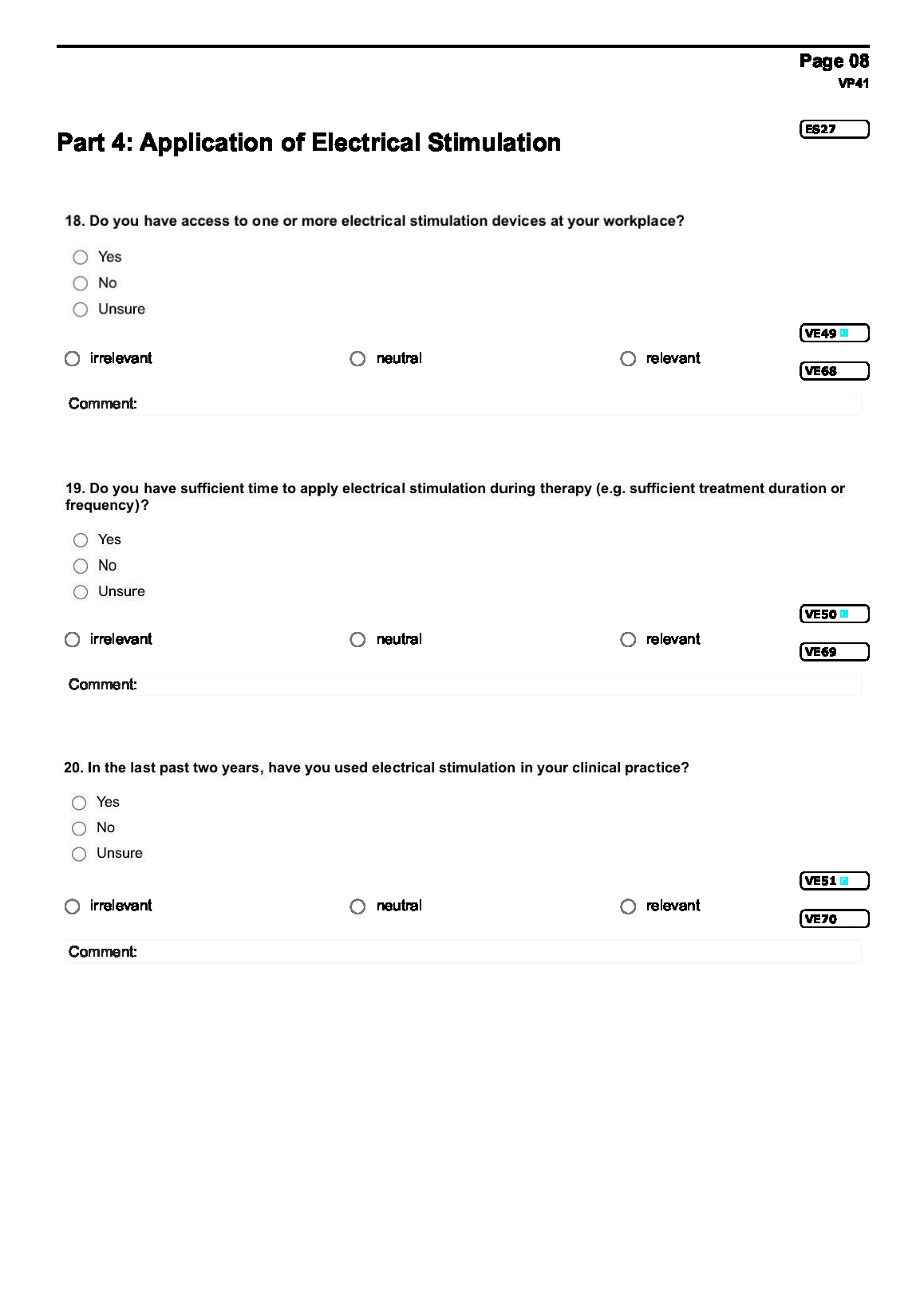

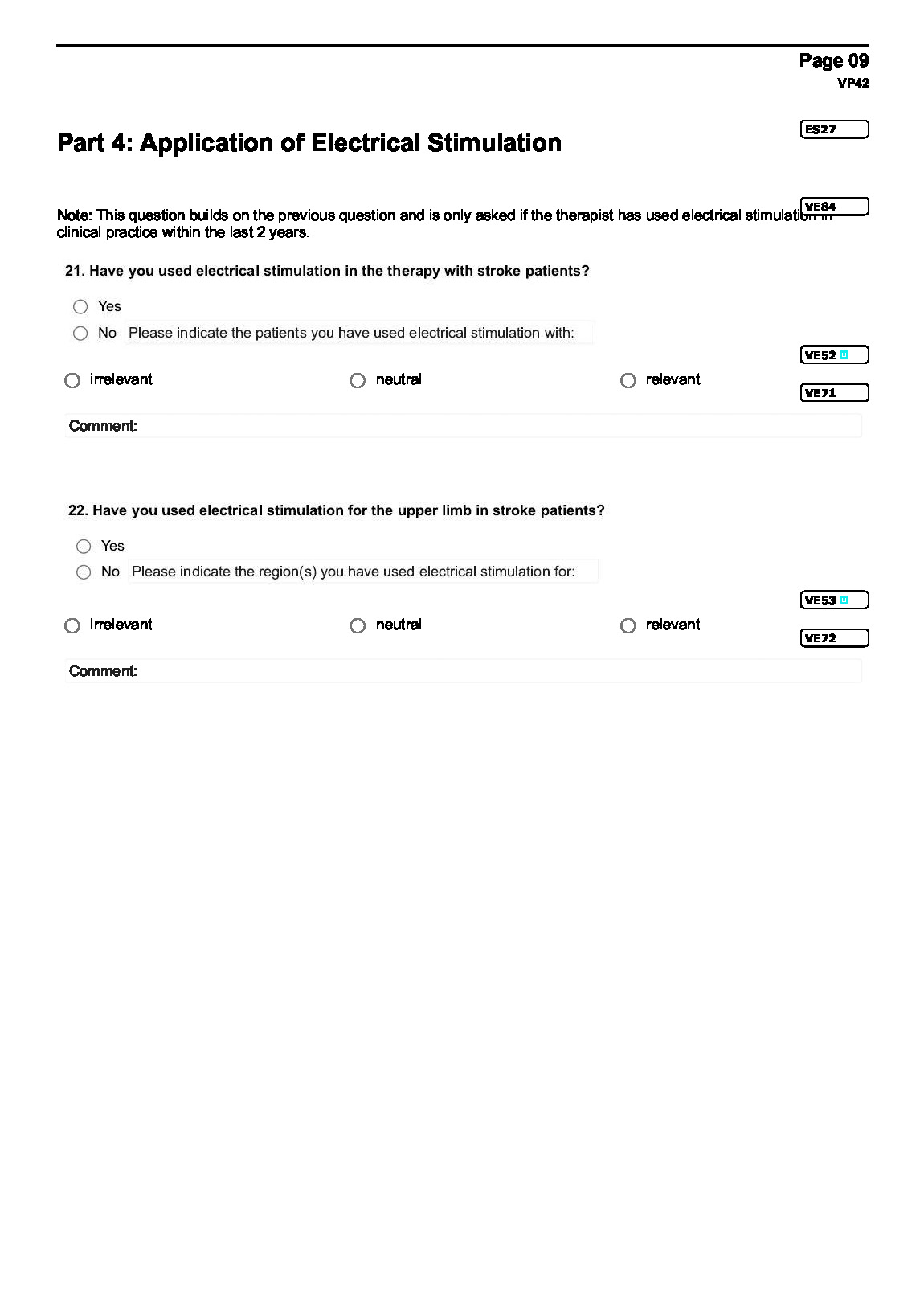

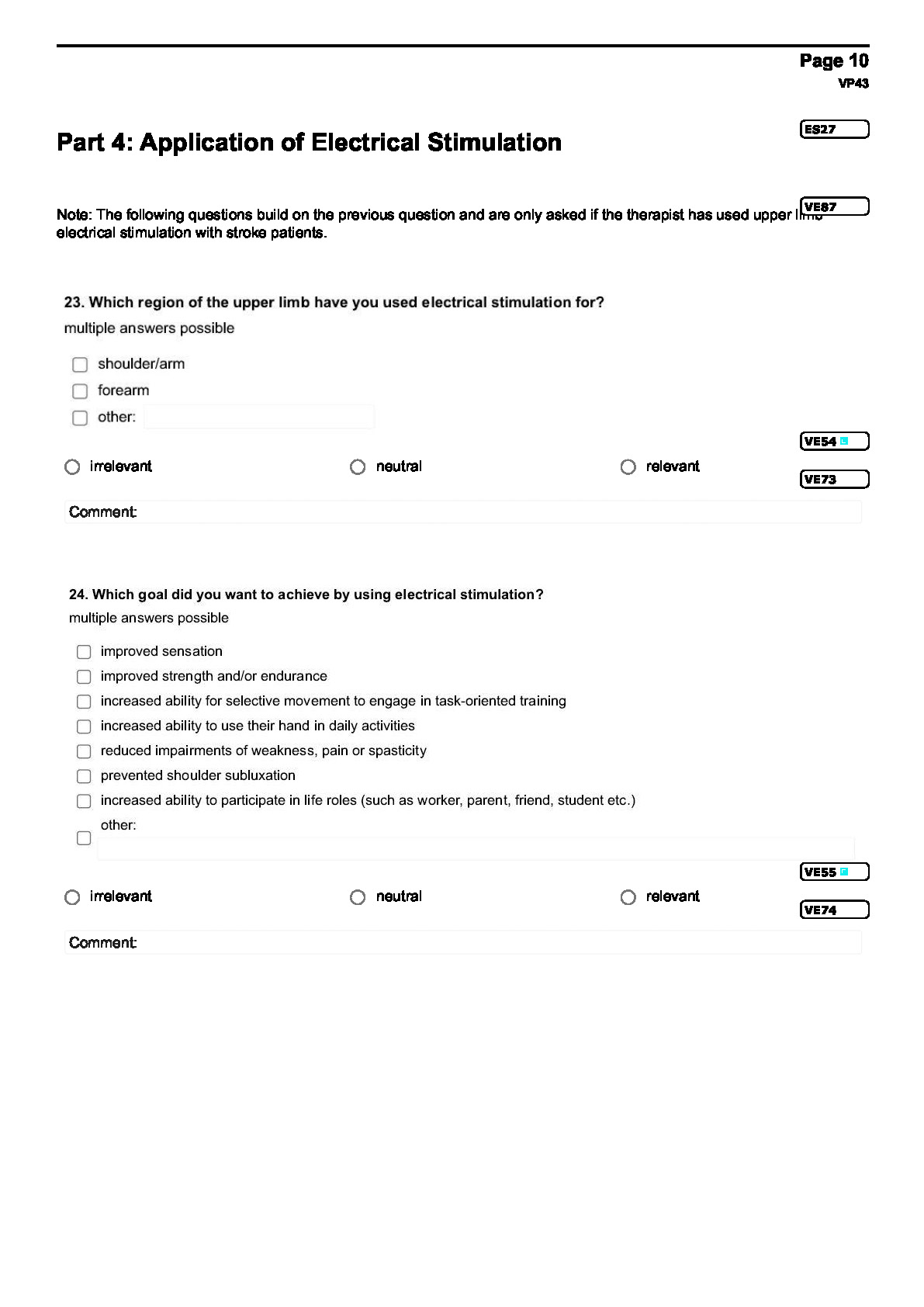


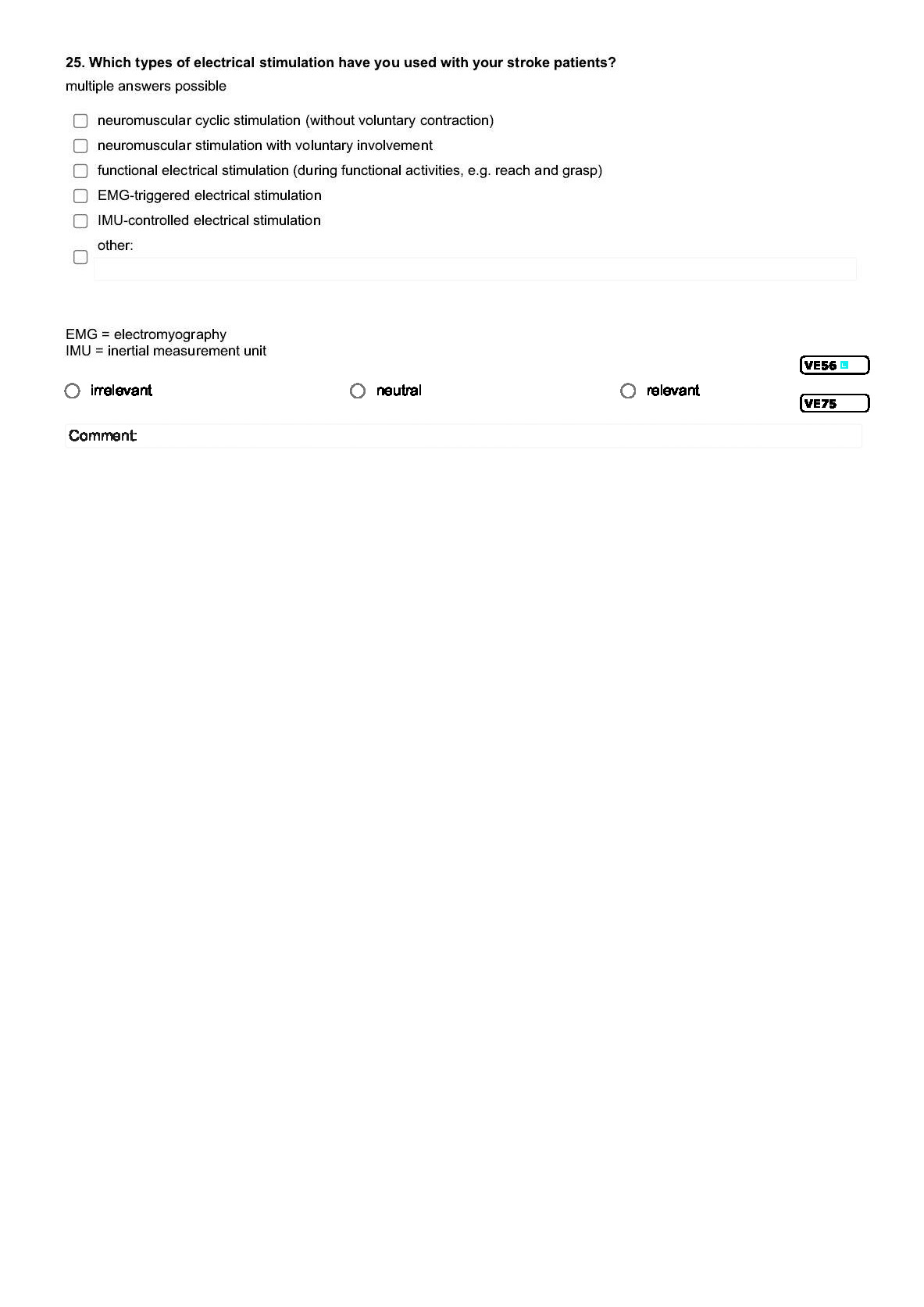


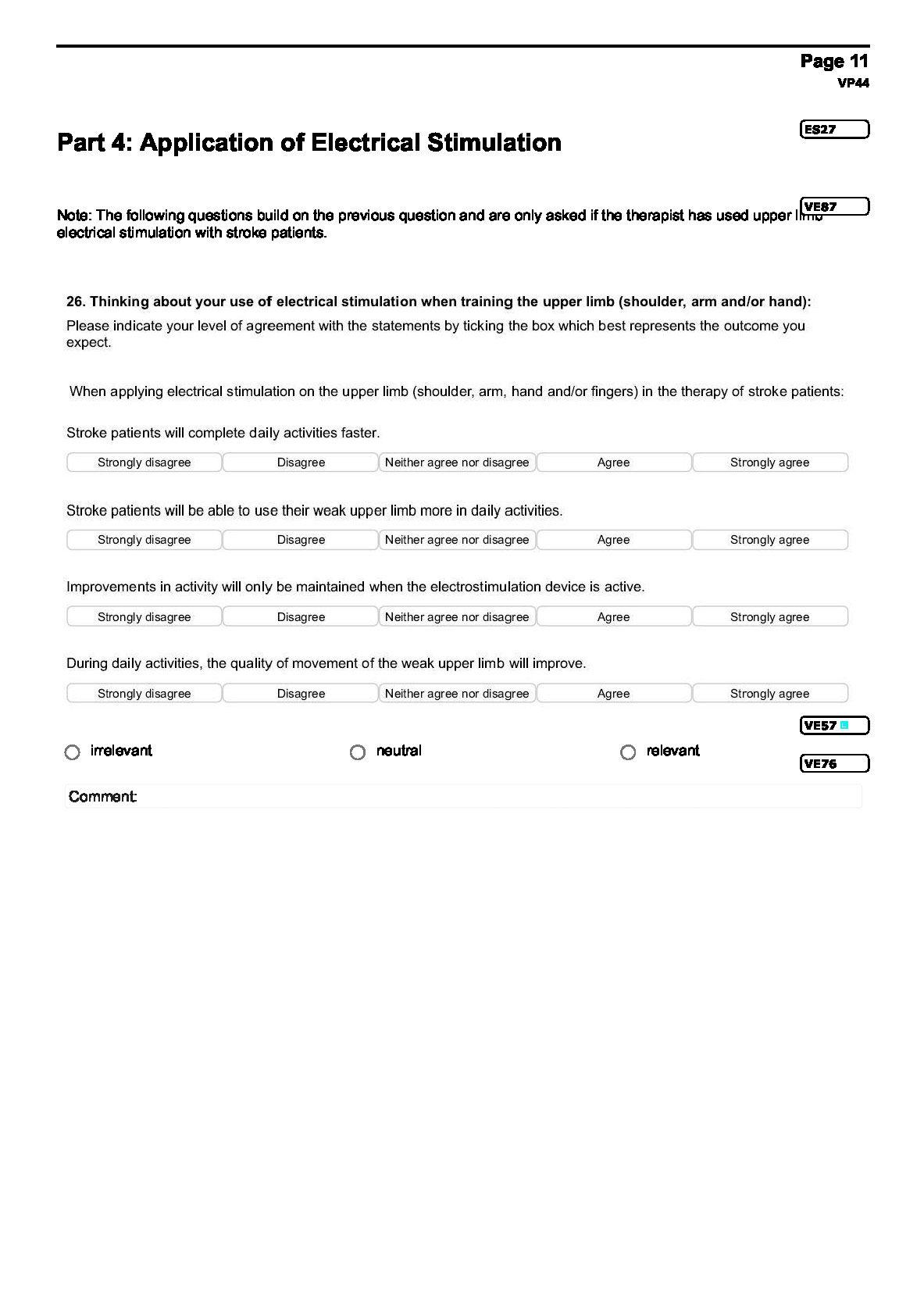

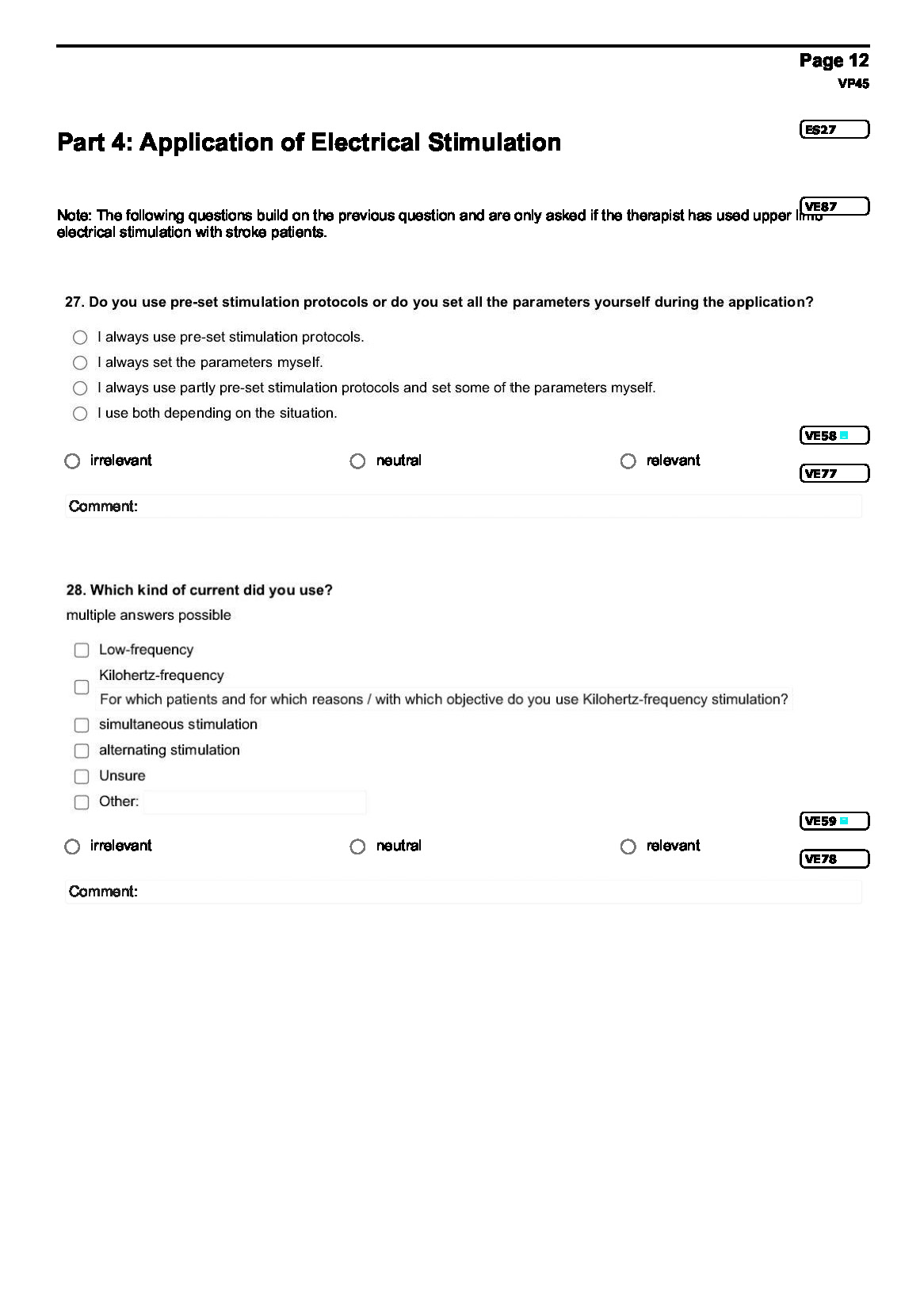


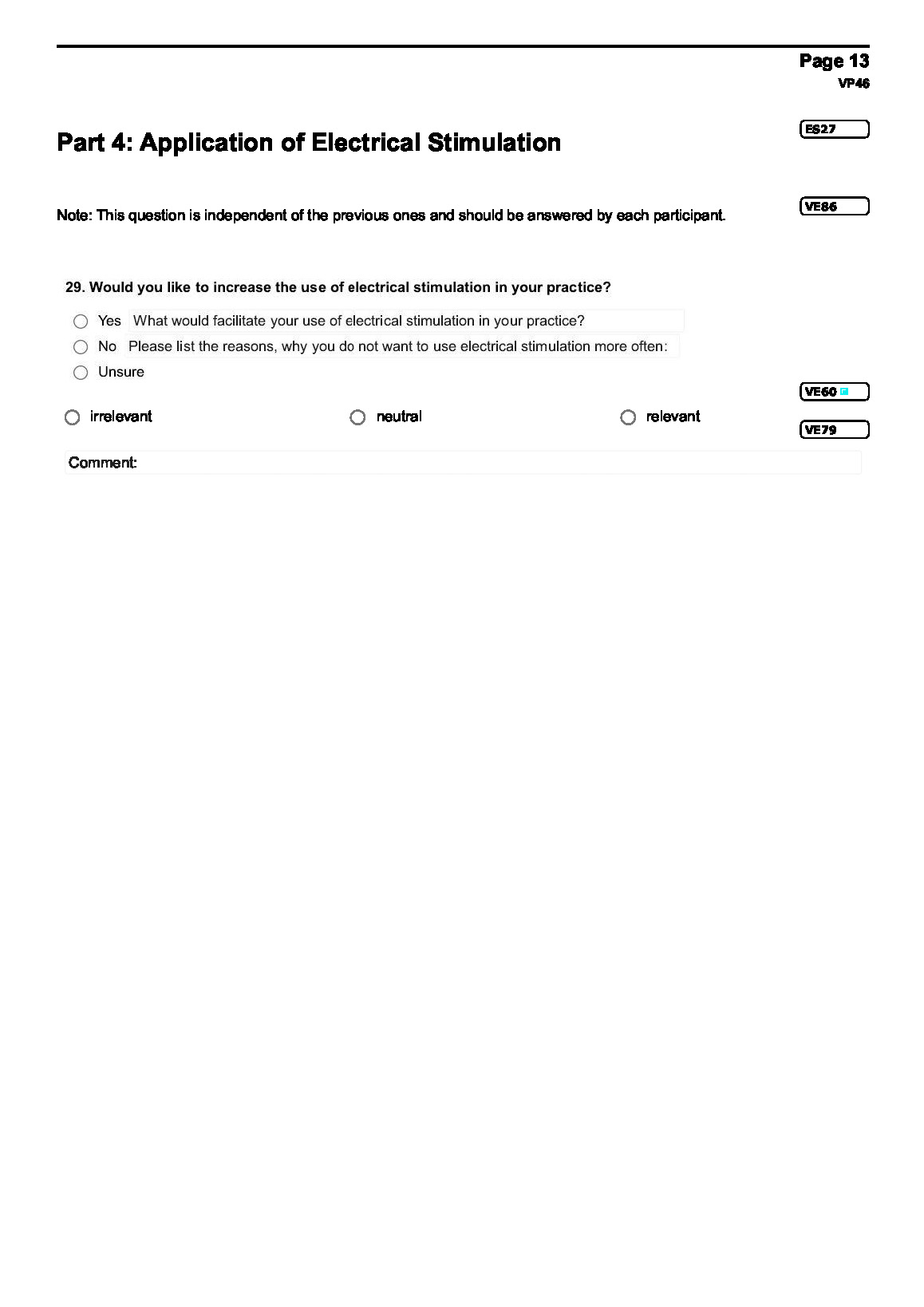


Figure S2: Validated German version of the survey


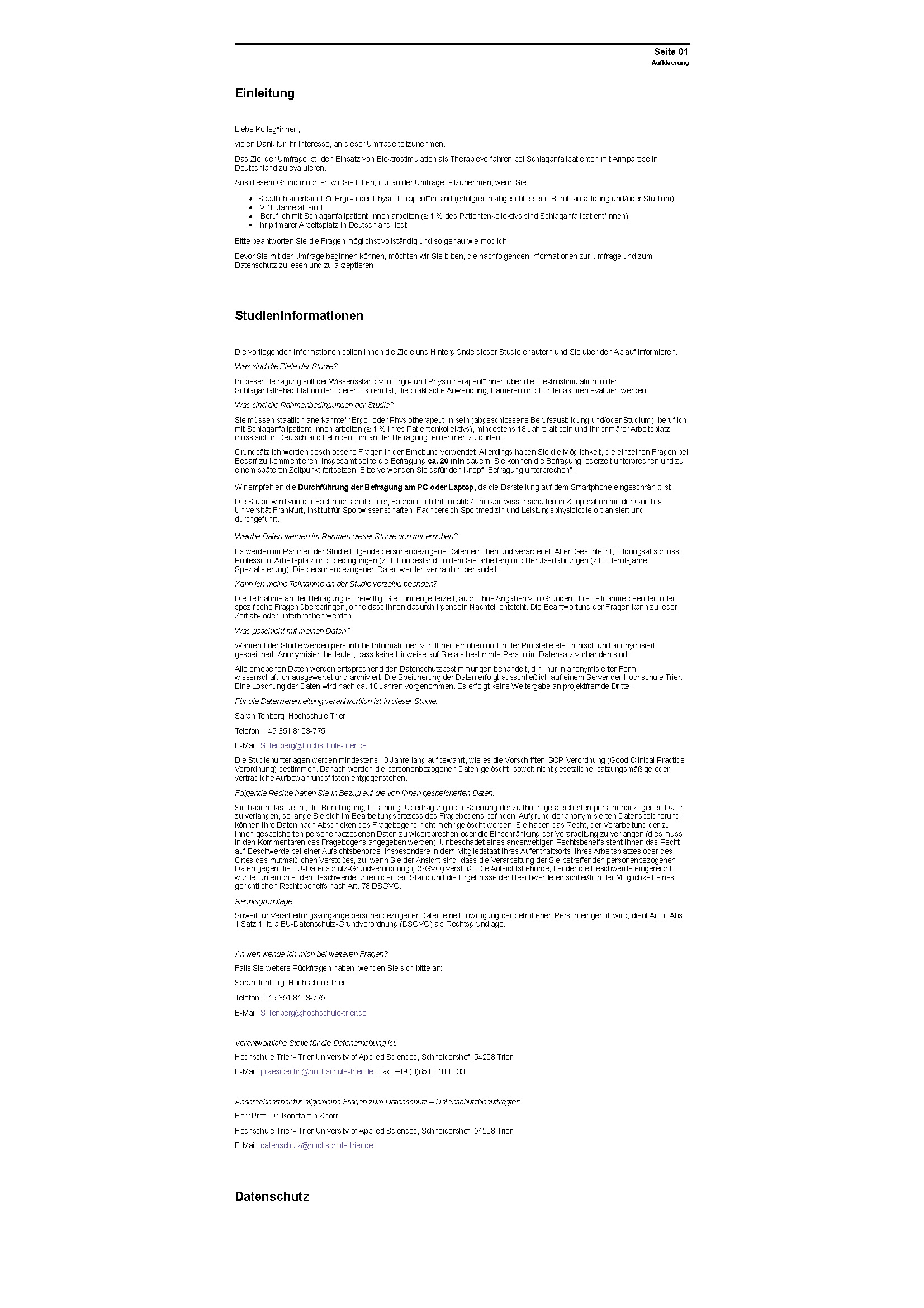


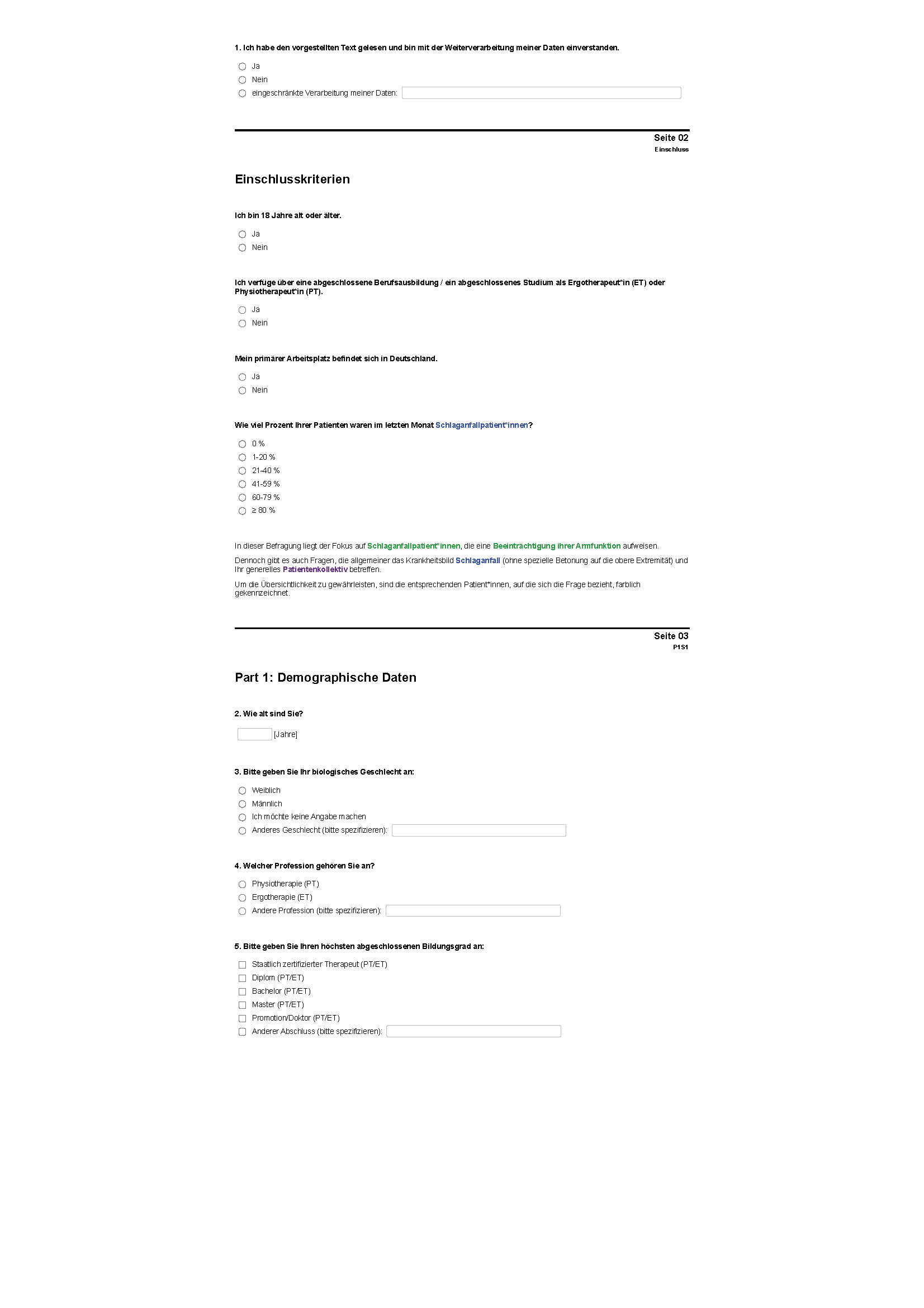

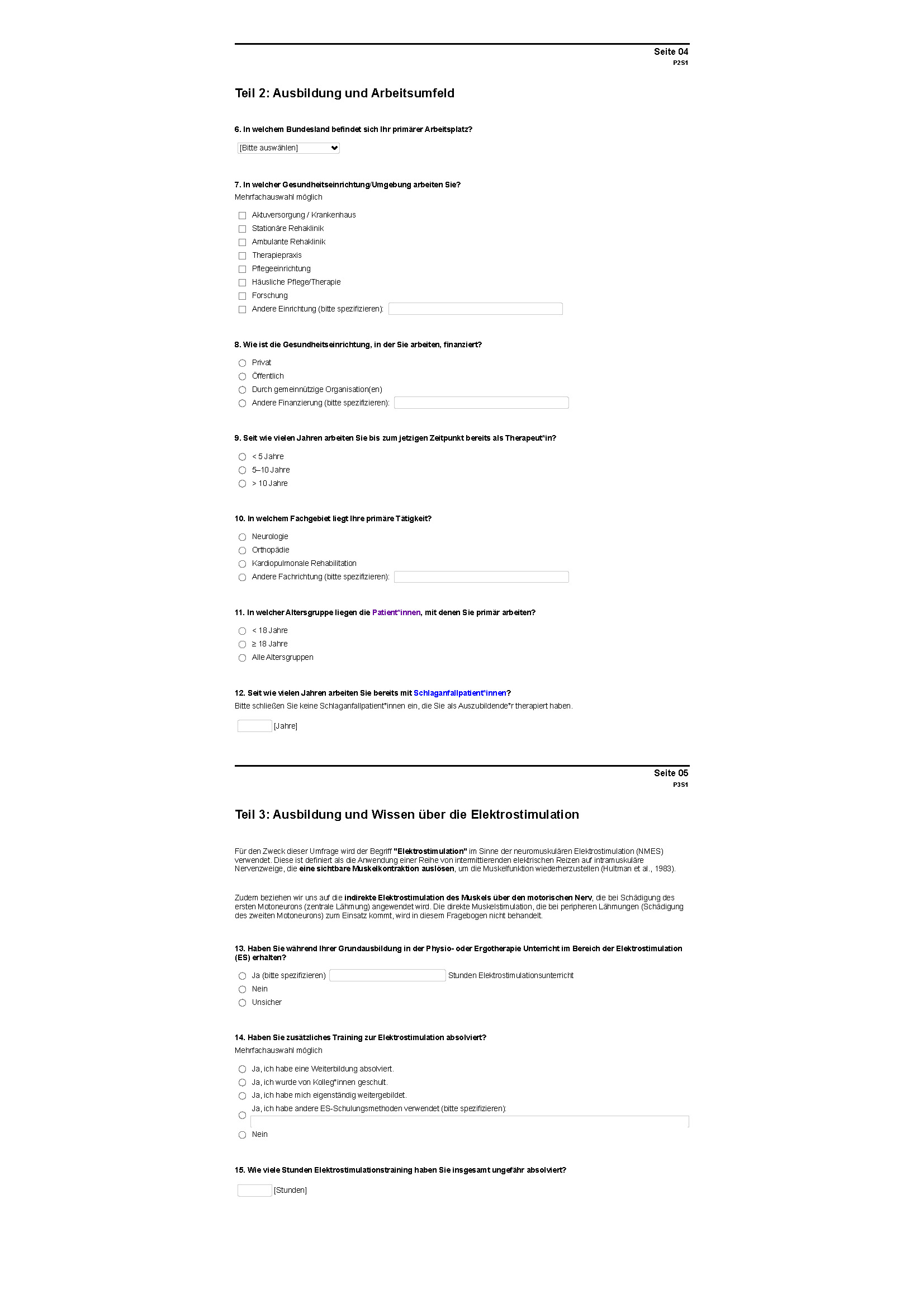

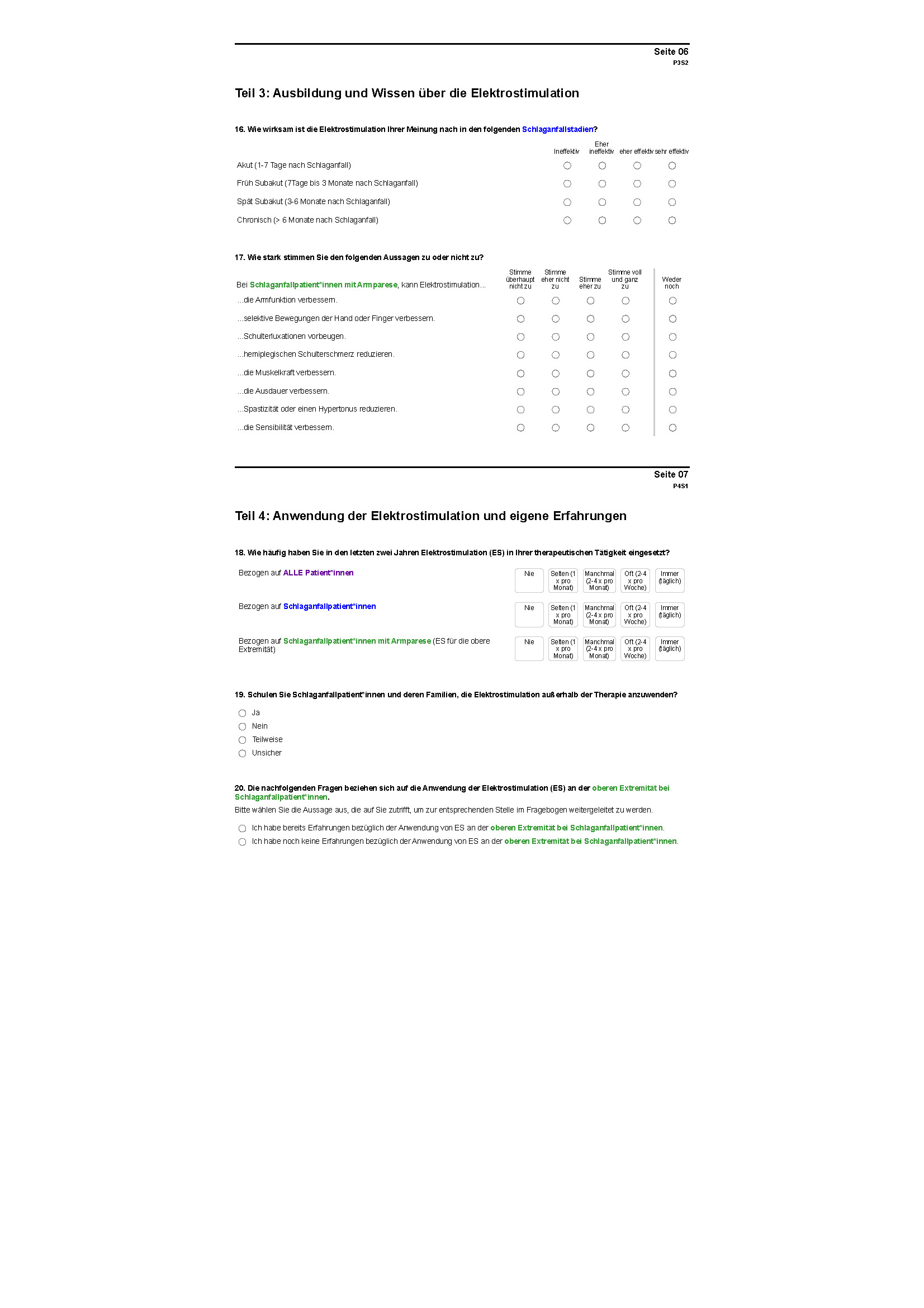

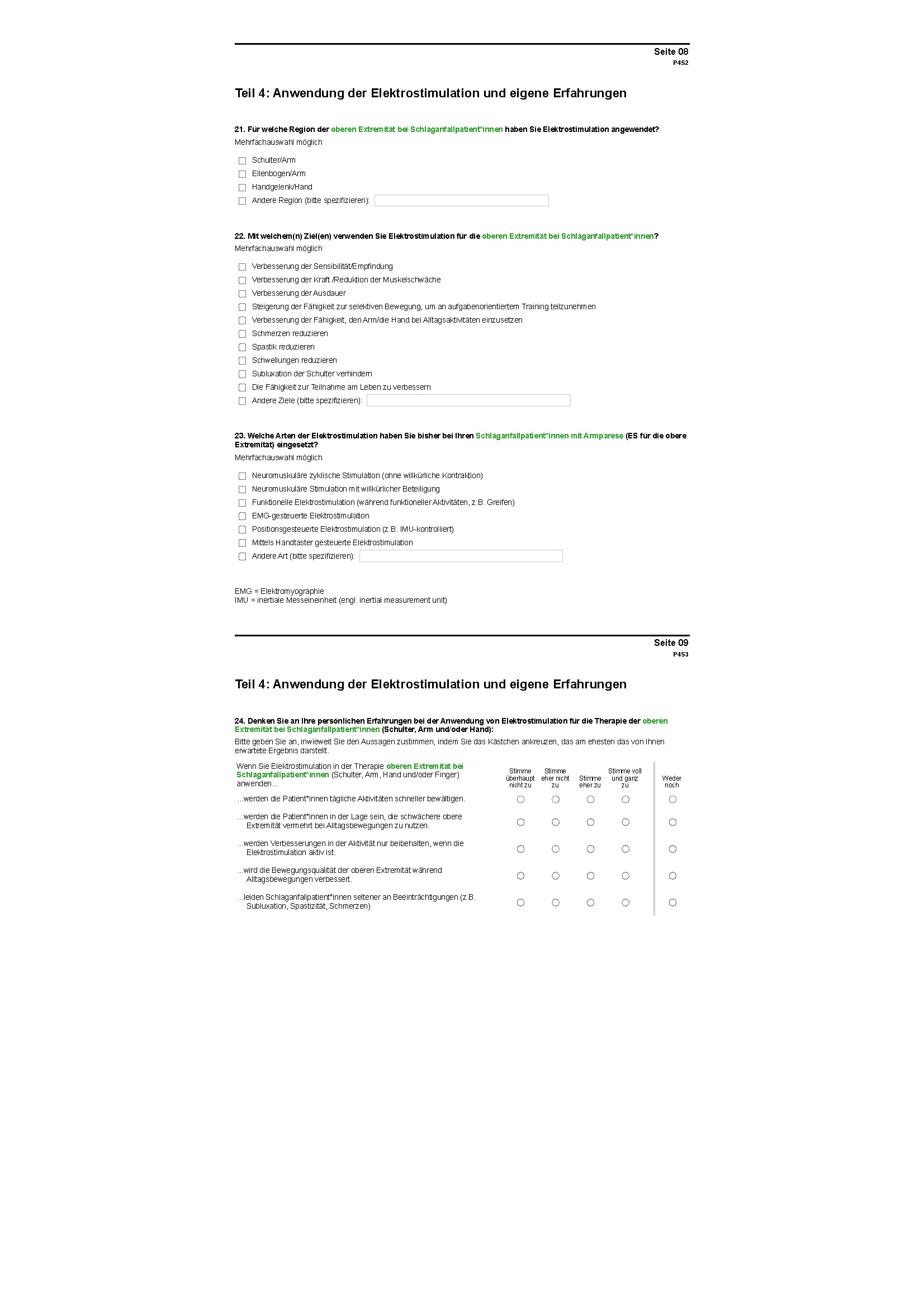

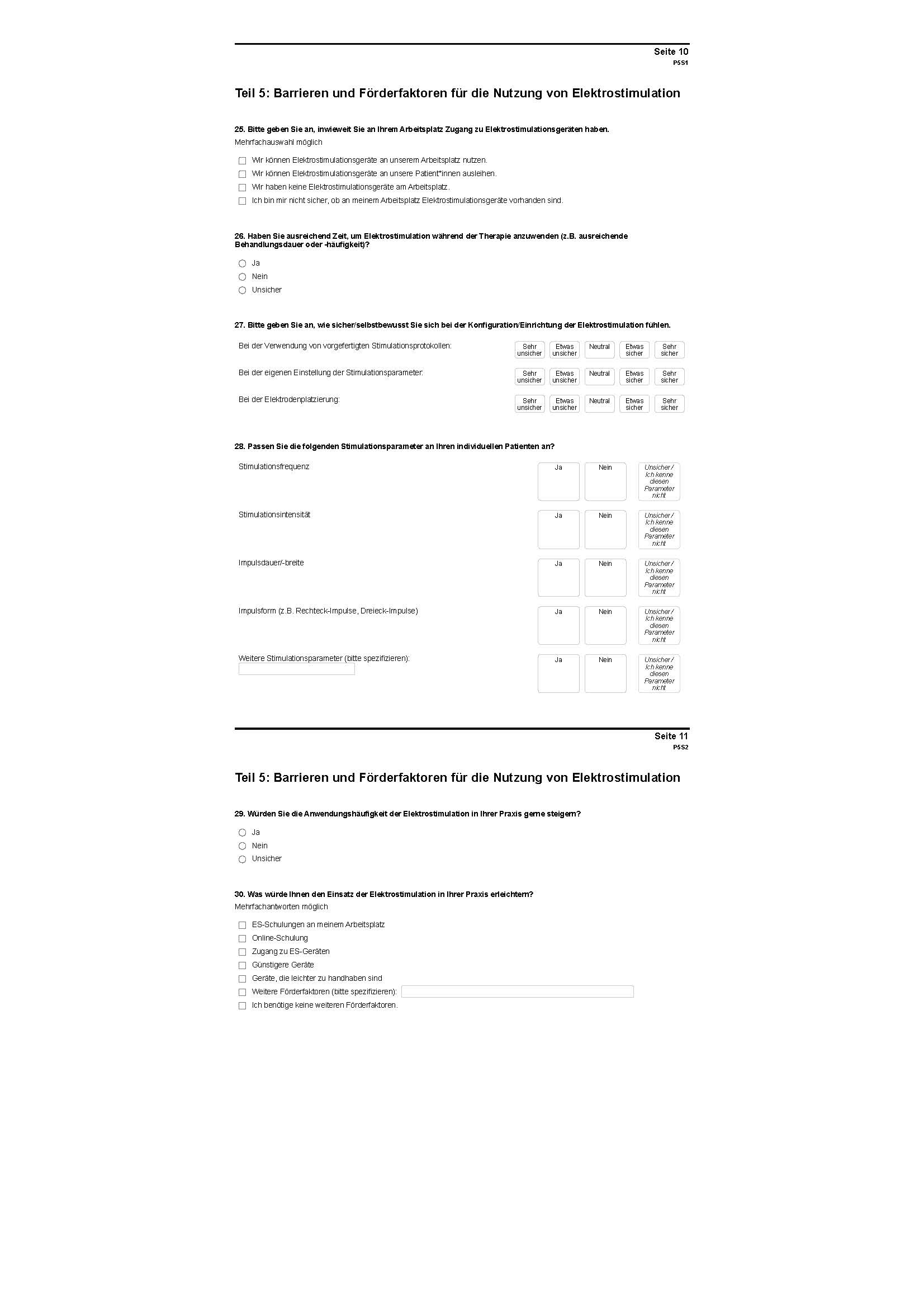

Supplement: Supporting Information — Figure S1: Validation version of the survey. Figure S2: Validated German version of the survey. [file 4697720.f1.docx]
